# Adaptive photoluminescence through a bioinspired antioxidative mechanism†

**DOI:** 10.1039/d4sc06096b

**Published:** 2024-10-09

**Authors:** Tobias Rex, Sebastian Baumert, Alexander Hepp, Gustavo Fernández, Cristian A. Strassert

**Affiliations:** a Universität Münster, Institut für Anorganische und Analytische Chemie Corrensstraße 28/30 48149 Münster Germany ca.s@uni-muenster.de; b Universität Münster, CeNTech, CiMIC, SoN Heisenbergstraße 11 48149 Münster Germany; c Universität Münster, Organisch-Chemisches Institut Corrensstraße 36 48149 Münster Germany fernandg@uni-muenster.de

## Abstract

Transition metal complexes are archetypal luminescent probes that are widely used for various applications ranging from optoelectronics to biomedicine. However, they face significant challenges such as photobleaching and photooxidative stress, which limit their performance. Herein, we introduce a photosystem-inspired concept based on the use of a vitamin (ascorbic acid, Asc-Ac) to adaptively suppress photobleaching of molecular luminophores. As a proof-of-concept compound, we have selected a new bis-cyclometalated Pt(II) complex (Pt-*t*Bu) and investigated its adaptive photoluminescence resulting from singlet dioxygen (^1^O_2_) photoproduction in the presence of Asc-Ac. Interestingly, the excited state quenching and subsequent photobleaching of Pt-*t*Bu in aerated solutions is suppressed by addition of Asc-Ac, which scavenges the ^1^O_2_ photosensitized by Pt-*t*Bu upon irradiation and results in an adaptive oxygen depletion with enhancement of luminescence. The adaptation is resilient for successive irradiation cycles with oxygen replenishment, until peroxidation overshooting leads to the degradation of Pt-*t*Bu by formation of a dark Pt(iv) species. The complexity-related adaptation with initial overperformance (luminescence boost) relies on the external energy input and cascaded feedback loops, thus biomimicking inflammation, as the repeated exposure to a stressor leads to a final breakdown. Our antioxidative protection mechanism against photobleaching can be successfully extended to multiple coordination compounds (*e.g.*, Ir(iii), Ru(ii) and Re(i) complexes), thus demonstrating its generality. Our findings broaden the scope of molecular adaptation and pave the way for enhancing the stability of molecular luminophores for multiple applications.

## Abstract 2

We introduce a bioinspired antioxidative mechanism to adaptively control the photoluminescence and prevent photobleaching of molecular phosphors.

## Graphical abstract

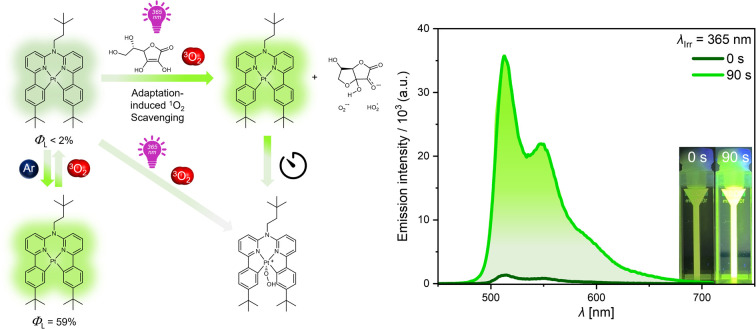

## Introduction

Biological systems employ diverse protective mechanisms to counteract degradation processes. A notable example is found in photosynthetic organisms,^1^ where the generation of singlet dioxygen (^1^O_2_) upon light irradiation with subsequent formation of triplet chlorophyll may lead to structural damage. To mitigate this threat, chloroplasts make use of defense mechanisms that involve scavenging of ^1^O_2_ by antioxidants such as β-carotene and α-tocopherol.^1^ Another prominent antioxidant is ascorbic acid (Asc-Ac; vitamin C), which neutralizes ^1^O_2_ to produce dehydroascorbate and H_2_O_2_, further illustrating the role of natural antioxidants in protecting cells from oxidative stress.^2–5^ While such antioxidative mechanisms are commonplace in biological systems, their role in artificial counterparts remains elusive.

A long-standing challenge in chemical systems that would greatly benefit from protective measures is the phenomenon of photobleaching.^6^ Akin to biological systems, molecular luminophores are significantly affected by oxidative stress, which can modulate their properties and function.^7–9^ Despite their widespread interest and versatile applications in optoelectronics,^10–14^ bioimaging^15–18^ and photocatalysis,^19^ transition metal complexes (including Ru(ii), Ir(iii) and Pt(ii) coordination compounds) are among the luminophores that suffer from photobleaching mainly due to Dexter energy transfer to triplet dioxygen, *i.e.*, quenching by ^3^O_2_ to yield highly reactive ^1^O_2_.^20–23^ As a result, they can be used as photosensitizers (PS)^24–26^ in photodynamic therapy (PDT).^27,28^ A prominent molecular design strategy to improve the performance and stability of Pt(ii) complexes lies in the use of tetradentate bis-cyclometallated ligands, which enable a stabilizing rigidification of the coordination environment.^29–31^ However, if quenching is not suppressed by deoxygenation, photooxidative stress triggered by irradiation with ^1^O_2_ generation ultimately leads to degradation.^32–34^ In recent years, various strategies have been developed to improve photostability through molecular design.^35^ These approaches include increasing the oxidation potential of molecules,^36^ introducing structural changes in the auxiliary ligand^37^ and creating supramolecular architectures such as micelles^38^ to enhance photostability. Additionally, the linking of protective agents^39^ or the embedding of luminophores in frameworks^40,41^ as well as the use of chelate complexes^42^ are effective methods for protection against chemical degradation. However, these strategies often alter the emission properties and the chemical environment of the luminophores. Therefore, there is a substantial need to develop alternative chemical approaches to stabilize luminophores in solution against oxidative stress while preserving their optical properties.

**Fig. 1:**
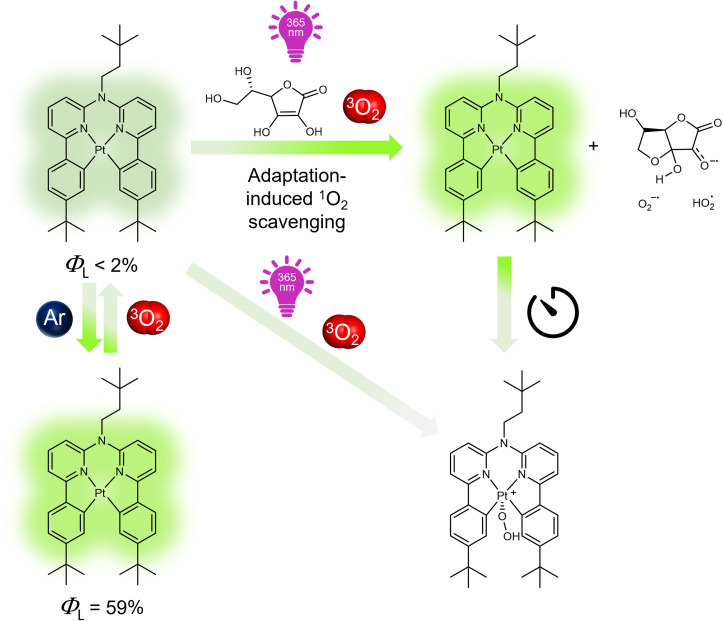
Structural formula of Pt-*t*Bu and schematic representation of its photoluminescence changes in response to light upon deoxygenation with Ar followed by equilibration with air, in the presence and absence of Asc-Ac. Feedback loops and light-driven equilibrium displacement enables the adaptation to the stressor (^3^O_2_) through ^1^O_2_ scavenging.

Herein, we introduce a bioinspired antioxidative strategy that adaptively suppresses quenching and prevents photobleaching of photosensitizing luminophores. To demonstrate our approach, we synthesized a Pt(ii)-based luminophore bearing a tetradentate bis-cyclometalated ligand (Pt-*t*Bu, Fig. 1)^43,44^ and examined its adaptive response to irradiation in the presence of ^3^O_2_ and Asc-Ac (for synthesis and characterization, see Fig. S2†). The three peripheral *tert*-butyl groups of Pt-*t*Bu were included to prevent aggregation, while the square-planar coordination geometry remains accessible to dioxygen. Pt-*t*Bu exhibits an intense photoluminescence in liquid solutions upon deoxygenation, but it rapidly undergoes photobleaching if quenched by dissolved ^3^O_2_ (Fig. 1). Interestingly, Asc-Ac scavenges the photosensitized ^1^O_2_ generated by Pt-*t*Bu, leading to a significant luminescence enhancement due to the depletion of ^3^O_2_. Re-equilibration with oxygen, followed by successive irradiation cycles, ultimately leads to the peroxidation of the Pt-*t*Bu to a Pt(iv) species. Hence, the initial overperformance followed by breakdown upon repeated aggression resembles stress-adaptation of organisms leading to collapse, irreparable damage or chronic conditions, such as rheumatoid arthritis or other autoimmune diseases.^45^

## Results

**Fig. 2:**
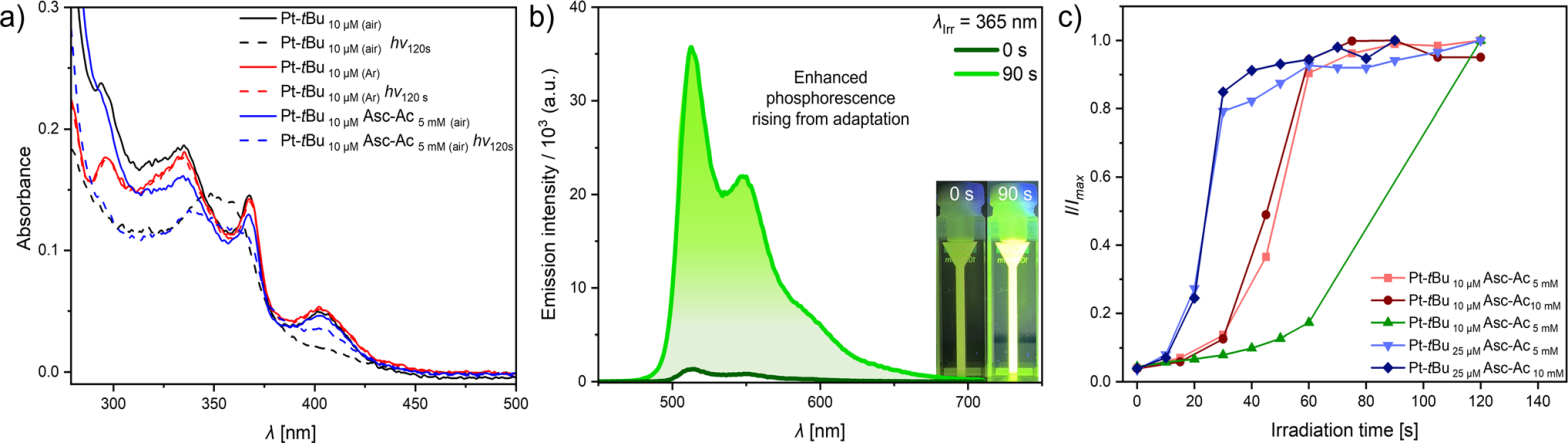
(a) UV-vis absorption spectra of Pt-*t*Bu in air-equilibrated DMF with (blue) and without (black) Asc-Ac, as well as of an Ar-purged (red) solution untreated (solid) and directly after irradiation (120 s) at 365 nm (dashed). (b) Exemplary photoluminescence spectra (Pt-*t*Bu 10 μM and Asc-Ac 5 mM) before and after prolonged irradiation in a photoreactor. Inset: pictures of the cuvette before and after the irradiation process in a photoreactor (*λ*_ex_ = 365 nm). (c) Plot of *I*/*I*_max_*vs.* irradiation time at different concentrations of Pt-*t*Bu and Asc-Ac at room temperature in DMF.

The UV-vis absorption and photoluminescence spectra of Pt-*t*Bu in organic solvents, such as dichloromethane (DCM) and dimethylformamide (DMF), show the characteristic photophysical properties of Pt(ii) complexes with tetradentate ligands (Table 1 and Fig. S34 and S35†).^43^ The higher-energy absorption bands below 350 nm are attributed to transitions into ^1^π–π* configurations, indicative of ligand-centered electronic states. Conversely, the lower-energy absorption bands appearing above 350 nm are associated with transitions into ^1^LC (ligand-centered) and ^1^MLCT (metal-to-ligand charge transfer) states. The photoluminescence spectra in DMF exhibit a maximum emission peaking at 514 nm, accompanied by a vibrational shoulder at 549 nm (metal-perturbed LC triplet state, *i.e.*, ^3^MP-LC, Fig. 2b and S34†). The substantial differences in the quantum yields and excited state lifetimes observed before and after deoxygenation (Table 1) demonstrate the significant quenching effect by ^3^O_2_.

**Table 1:** Summary of photophysical data for Pt-*t*Bu

	*Φ*_L(air)_ (±2) (%)	*Φ*_L(Ar)_ (±3) (%)	*τ*_(air)_ (ns)	*τ*_(Ar)_ (ns)
Pt-*t*Bu	<2	59	124.6 ± 0.3	6895 ± 9

The phosphorescence of Pt-*t*Bu is quenched by ^3^O_2_ in a diffusion-controlled exciplex formation (Dexter energy transfer), leading to the production of ^1^O_2_. In fact, the phosphorescence of ^1^O_2_ becomes evident in the NIR (peaking at 1275 nm, Fig. S37†).

To assess the potential degradation of Pt-*t*Bu due to photooxidative stress, we examined its stability under UV irradiation (*λ*_ex_ = 365 nm) by measuring the UV-vis absorption spectra and the photoluminescence intensity (Fig. S35 and S36†). Subsequent irradiation of Pt-*t*Bu solutions at two different concentrations (10 μM and 25 μM) with UV light in a photoreactor revealed that Pt-*t*Bu undergoes partial decomposition in the presence of ^3^O_2_. This was verified by the loss of the ^1^MLCT band at 400 nm in the UV-vis spectra and the drop of emission intensity in the photoluminescence spectra (Fig. 2a, S35 and S36†). On the other hand, Pt-*t*Bu exhibits resistance against UV irradiation upon deaeration, as evident from its invariant absorption and emission features (Fig. 2a, S38 and S39†). These results confirm that photosensitized ^1^O_2_ is the primary cause for the photobleaching of Pt-*t*Bu.

To study the biomimetic protection involving an antioxidant, we prepared a mixture that combines Asc-Ac and Pt-*t*Bu. In DMF, an excess of Asc-Ac in the presence of Pt-*t*Bu under UV irradiation (in a photoreactor) indicated that the stressor (^3^O_2_ photoconverted into ^1^O_2_) no longer affects the luminophoric PS Pt-*t*Bu. Hence, only minor changes were observed in the MLCT absorption band before and directly after long-term irradiation (120 s, Fig. 2a and S40†).

The changes in the higher energy absorption bands (<400 nm) can be attributed to the formation of H_2_O_2_, dehydroascorbate, and a new coordination-chemical species (Fig. 1 and S40†). Furthermore, a narrowing of these bands becomes evident upon aging after irradiation (Fig S40,† right), which is accompanied by a decrease in the MLCT band located around 400 nm, which can be associated to the formation of a Pt(iv) species. Herein, a hydroperoxo ligand is incorporated upon oxidation of the former Pt(ii) complex, thus influencing both absorption and emission spectra. Remarkable, though, was the immediate boost of the emission intensity and the prolonged excited state lifetime of Pt-*t*Bu (Fig. 2b and S49†).

To understand this behavior, we explored the influence of gradual irradiation on the emission intensity of Pt-*t*Bu at two different concentrations (10 μM and 25 μM), in order to double the ^1^O_2_ photoproduction rate, in the presence of Asc-Ac at two different concentrations (5 mM and 10 mM) (Fig. S41 and S42†). It was ensured that there was no gaseous headspace for the air-equilibrated system, as otherwise external oxygen could re-diffuse into the solution. Notably, the phosphorescence intensities increased until they reached a plateau, indicating that the Pt-*t*Bu is no longer quenched by ^3^O_2_ (Fig. 2c). In addition, we observed that both the concentration of Pt-*t*Bu and the irradiation time (10 s and 15 s) influence the rate of adaptation. A higher concentration of Pt-*t*Bu results in a reduced lag time to reach the plateau (Fig. 2c). We attribute this phenomenon to an increased ^1^O_2_ production rate and its trapping by the excess of Asc-Ac at higher concentrations of Pt-*t*Bu. This suggests that ^3^O_2_ is converted to ^1^O_2_ and subsequently neutralized by Asc-Ac to form dehydroascorbate. In contrast, decreasing the irradiation intervals from 15 s to 10 s at a constant concentration (10 μM) resulted in a significantly delayed intensity rise (Fig. 2c). Thus, a substantial enhancement in emission can only be achieved upon extended irradiation (60 s). This can be explained by the slower oxygen photoactivation and reaction with Asc-Ac. On the other hand, we observed that an increase in the concentration of Asc-Ac from 5 mM to 10 mM had no effect on the onset. Note that the most efficient protection of the luminophore occurs using 200–1000 equivalents of ascorbic acid to ensure the quenching of the ^1^O_2_ produced during the irradiation process. Moreover, plotting (*I*/*I*_max_) − 1 *vs.* increasing quencher concentration (*i.e.*, *vs.* 1 − (*t*/*t*_max_), meaning progressively shorter irradiation times) revealed that the process can be represented in a Stern–Volmer-like plot (Fig. S43†). Besides the intensity changes, the lifetimes after reaching the plateau were measured. For Pt-*t*Bu (10 μM) with Asc-Ac (5 mM), the lifetime increased to *τ* = 5.0 μs after irradiation (120 s). This contrasts with the shorter lifetime of *τ* = 120 ns observed for Pt-*t*Bu in air-equilibrated solutions (Fig. S47 and S49†), confirming the partial scavenging of the quencher (^3^O_2_). Nevertheless, it does not reach the lifetime upon full deoxygenation (*vide infra*), indicating the involvement of other quenching phenomena or residual ^3^O_2_. This is also evidenced by the fluctuations within the plateau phase.

**Fig. 3:**
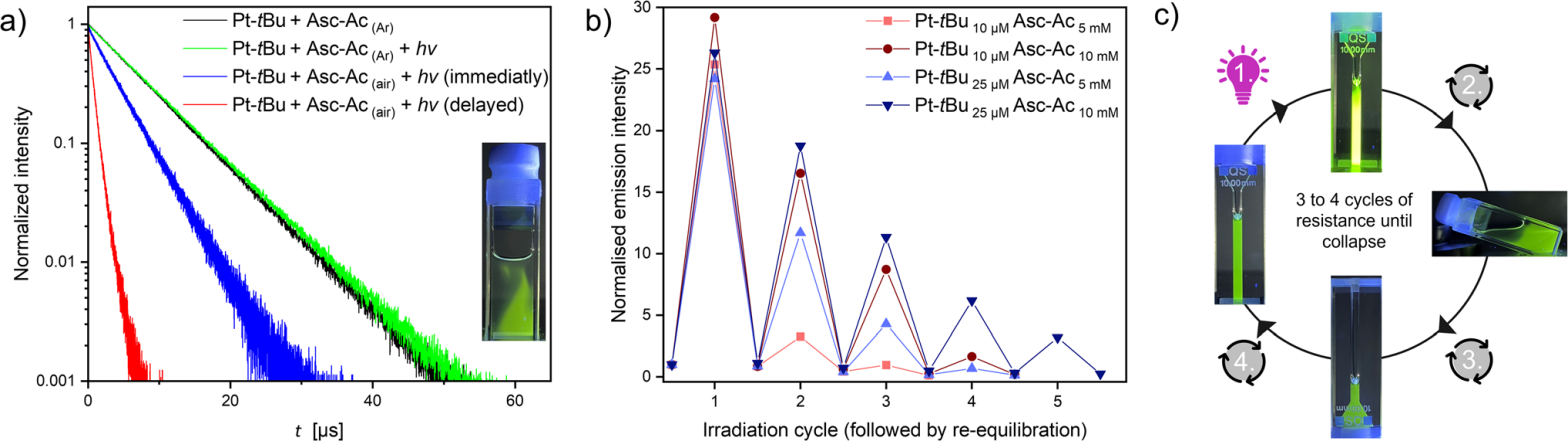
(a) Immediate (blue) and delayed (red) luminescence lifetime measurement (monitored at 514 nm) of the mixture (Pt-*t*Bu 25 μM and Asc-Ac 10 mM with an air headspace) after the irradiation process in the photoreactor; comparison with Ar-purged mixtures before (black) and after irradiation (green). Inset: picture of the air-equilibrated cuvette shortly after irradiation under UV light (*λ*_ex_ = 365 nm). (b) Plot of the emission intensity at 514 nm *vs.* the number of irradiation cycles at different concentrations of Pt-*t*Bu and Asc-Ac (with air headspace) at room temperature in DMF. Each cycle consists of irradiation in the photoreactor (*λ*_ex_ = 365 nm) and subsequent re-equilibration with the headspace by shaking the cuvette. The system shows a resistance against stressor replenishment but collapses after multiple re-equilibrations by the formed products. (c) Schematic illustration of an irradiation cycle under UV light (*λ*_ex_ = 365 nm).

After a full irradiation cycle (90 s) of the sealed air-equilibrated mixture (identical to those in Fig. 2b) with a waiting period of one hour, the traces of re-diffused oxygen can be again scavenged, achieving the previous plateau yet without substantial photobleaching, as indicated by the minimal change in the MLCT band (Fig. S44,† inset). We hypothesize that the observed drop after three iterations may be associated with the depletion of Asc-Ac resulting in the slower kinetics due to a constant trigger. To ascertain that oxygen is indeed the predominant factor mediating the process and to rule out the formation of other species such as Pt-*t*Bu-Asc-Ac, we conducted experiments upon Ar-purging and visually tracked the re-diffusion of minute oxygen traces into the system. The lifetimes of the Ar-purged samples, both with and without Asc-Ac, were identical (Fig. S48 and S51†). Notably, after irradiating the Ar-purged mixture yet including Asc-Ac, even the last traces of oxygen are evidently scavenged (Fig. 3a, S51 and S52†) while maximizing the lifetime.

Contrastingly, in an open system (*i.e.*, with air headspace), the significant drop of lifetime upon passive oxygen re-equilibration was confirmed by lifetime measurements at different recovery times. Monitoring irradiation of the system in the photoreactor, followed by immediate and delayed (2 min) lifetime measurements, a drastic shortening was observed (Fig. 3a). This rapid re-equilibration of the system through passive oxygen diffusion (after the quencher has been scavenged) illustrates the dynamic nature of the process. Furthermore, the intensity gradient at the interface can be visually observed under UV light (inset Fig. 3a), confirming that the ^3^O_2_ is the central actor. To gain further insights into how sustained stress exposure affects the system's resilience, we systematically re-equilibrated the mixture after each irradiation cycle (90 s) with air (Fig. 3b and c).

We observed two significant features: the adaptation ability at each cycle (before complete breakdown) depends on the concentration of both Pt-*t*Bu and Asc-Ac. Increasing the concentration of Pt-*t*Bu and Asc-Ac leads to a longer stability of the system, which can be explained in two ways: Firstly, higher Pt-*t*Bu concentrations generate comparatively more ^1^O_2_ accompanied by an increased conversion of Asc-Ac before bleaching of Pt-*t*Bu can occur. Secondly, the conversion capacity increases with a higher Asc-Ac concentration, thereby hindering the reaction of Pt-*t*Bu with ^1^O_2_. However, after several cycles, the system undergoes degradation with exhaustion and observable depression due to permanent re-equilibration with the stressor. Mass spectrometry revealed that the photobleaching and the generated H_2_O_2_ results in the formation of a non-emissive Pt(iv) derivative of Pt-*t*Bu, wherein oxygen occupies an axial position (Fig. S33†). Overall, the response resembles initial performance boost of biological systems as a response to an aggressor, followed by fatigue and breakdown upon repeated or sustained exposure to harm.^46^ Hence, adaptation to stress with suppression of the stressor (^3^O_2_) causes an overperformance of the system (higher luminescence intensity), much like an organism immediately adapting to stress (adrenaline, short-term response) and coping with it in a sustained manner (cortisol, mid-term response); however, sustained exposure causes fatigue and failure.^47,48^

**Fig. 4:**
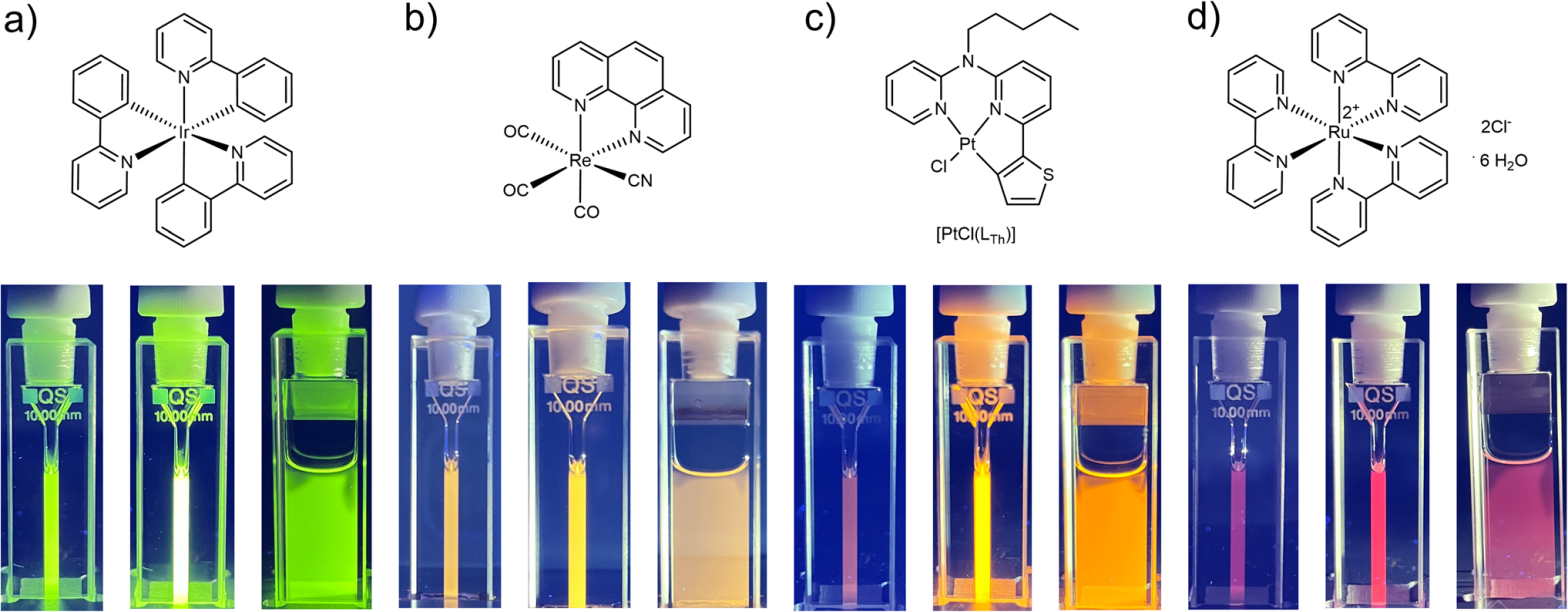
Structural formulae of *fac*-tris-(2-phenylpyridine)iridium(iii)^49^ (a), tricarbonyl(cyanido)(1,10-phenanthroline)rhenium(i) (b),^51^ [PtCl(L_Th_)]^24^ (c) and tris(2,2′-bipyridyl)ruthenium(ii) dichloride hexahydrate^50^ (d). Shown are the corresponding pictures of the cuvettes with each complex (25 μM) and Asc-Ac (10 mM) in DMF before and after the irradiation in a photoreactor (as observed under UV light, *λ*_ex_ = 365 nm). Pictures on the right demonstrate the diffusion of fresh oxygen from the headspace after irradiation.

To ultimately demonstrate the broad applicability of the concept, we subjected different photosensitizing coordination compounds (including Ir(iii),^49^ Ru(ii)^50^ and Re(i)^51^ complexes as well as another Pt(ii)^24^ species, 25 μM) to irradiation with Asc-Ac (10 mM) in DMF (Fig. 4). Remarkably, Asc-Ac scavenges the photosensitized ^1^O_2_ generated by the three PS, resulting in a significant increase in emission intensity upon adaptation in the respective emission maxima from green to red (Fig. 4a–c, middle cuvettes). The rapid re-equilibration of the system, driven by passive oxygen diffusion, can be visually observed at the interface (Fig. 4a–c, right cuvettes), which underlines the generality of this adaptation for all tested PS and demonstrates the broad scope of our antioxidative mechanism.

## Conclusion

In conclusion, we have developed a bioinspired strategy based on the use of vitamin C as the antioxidant to adaptively protect molecular phosphors from the long-standing limitation of photodegradation by molecular oxygen. As a proof-of-concept, we have focused on a new Pt(ii) complex, and the generality of our approach has been validated by examination of various types of coordination compounds. In air-equilibrated solutions, all luminophores undergo quenching and photobleaching. However, the addition of ascorbic acid with concomitant irradiation deactivates the stressor (molecular oxygen), and restores the phosphorescence. However, repeated exposure to the stressor for over several cycles ultimately surpasses the system's resilience. This demonstrated at chemical level how repeated stress exposure influences a system until it reaches exhaustion. It draws parallels to the immune response, where prolonged triggers can lead to pathological responses, shifting from persistent chronic inflammation to organ failure.^45^ Hence, oxidation of the Pt(ii) to a new Pt(iv) species mediates the breakdown, much like a dysregulated response in the immune system. Adaptation results from a complex array (here, multicomponent mixtures) that is kept out of equilibrium (by irradiation) while involving multiple feedback loops (*e.g.*, photosensitization and trapping of singlet dioxygen), followed by degradation of the photosensitizer (in this case, by the accumulation of less reactive peroxides). Our results pave the way for photoluminescence protection and broaden the scope of molecular adaptation^52^ to complex multicomponent mixtures.

## Author contributions

T. R. and C. A. S. designed the project. T. R. performed the synthesis, UV-Vis and photoluminescence studies. T. R. and A. H. conducted all NMR studies. T. R. and S. B. performed the photoirradiation studies. T. R., G. F. and C. A. S. prepared the first draft of the manuscript including the figures, which was then revised and adapted upon contribution from all authors. The overall project was supervised by G. F. and C. A. S.

## Conflicts of interest

The authors declare no competing interests.

## Supplementary Material

SC-015-D4SC06096B-s001

## Data Availability

The data supporting the findings of this study are provided in the ESI† and are available from the corresponding author on request.
