# Supplementary material for: Adaptive photoluminescence through a bioinspired antioxidative mechanism
Source: Chem Sci. 2024 Oct 9;15(45):18881–7. doi: 10.1039/d4sc06096b (PMC11515932; doi:10.1039/d4sc06096b)
Supplement: SC-015-D4SC06096B-s001 [file SC-015-D4SC06096B-s001.pdf]

Supplementary Information (SI) for Chemical Science.
This journal is © The Royal Society of Chemistry 2024

Electronic Supplementary Information

Adaptive photoluminescence through a bioinspired antioxidative mechanism

Tobias Rex,[a,b] Sebastian Baumert,[c] Alexander Hepp,[a] Gustavo Fernández,[c]* and Cristian A. Strassert[a,b]*

[a] Tobias Rex, Dr. Alexander Hepp, Prof. Dr. Cristian A. Strassert

Universität Münster, Institut für Anorganische und Analytische Chemie, Corrensstraße 28/30, 48149 Münster (Germany).

E-mail: ca.s@uni-muenster.de

[b] Tobias Rex, Prof. Dr. Cristian A. Strassert

Universität Münster, CeNTech, CiMIC, SoN, Heisenbergstraße 11, 48149 Münster (Germany).

[c] Sebastian Baumert, Prof. Dr. Gustavo Fernández

Universität Münster, Organisch-Chemisches Institut, Corrensstraße 36, 48149 Münster (Germany).

E-mail: fernandg@uni-muenster.de

Contents

1. Methods and materials 2

2. Synthesis and characterization 4

3. NMR and mass spectra 9

4. Photophysical characterization 24

5. Broadening the scope to other metal complexes 34

6. Supplementary References 35

1. Methods and materials

For all reactions, chemicals were used as purchased without further purification. For column chromatography, silica gel 60 (0.063 – 0.200 mm) was used, purchased from Merck.

1.1 NMR spectroscopy and mass spectrometry

NMR spectra were obtained at the Institut für Anorganische und Analytische Chemie (Universität Münster), using a Bruker AVANCE NEO/ Bruker AVANCE I/ Bruker AVANCE III (400 MHz) or a Bruker AVANCE NEO (500 MHz). All measurements were performed at 300 K unless mentioned otherwise. The ^{1}H-NMR and ^{13}C-NMR chemical shifts (δ) of the signals are given in parts per million and are referenced to the residual signal of the deuterated solvent. The signal multiplicities are abbreviated as follows: s, singlet; d, doublet; t, triplet; q, quartet; m, multiplet. ^{1}H-NMR chemical shifts are given relative to TMS and are referenced to the solvent signal. Spectra of other nuclides like ^{13}C are referenced according to the proton resonance of TMS as the primary reference for the unified chemical shift scale.[1] The signal multiplicities are abbreviated as follows: s, singlet; d, doublet; t, triplet; q, quartet; m, multiplet. All coupling constants (J) are given in Hertz (Hz). Exact mass (EM) determination by mass spectrometry (MS) was carried out at the Organisch-Chemisches Institut (Universität Münster) using a LTQ Orbitap LTQ XL (Thermo-Fisher Scientific, Bremen) with electrospray injection (ESI).

1.2 Photophysical characterization

Absorption spectra were measured with a Shimadzu UV-3600 I plus UV-VIS-NIR spectrophotometer. For all measurements, matched quartz (Hellma®) cuvettes were used. Photoluminescence quantum yields were measured with a Hamamatsu Photonics absolute PL quantum yield measurement system (C9920-02) equipped with a L9799-01 CW Xe light source (150 W), a monochromator, a C7473 photonic multi-channel analyser, an integrating sphere and employing U6039-05 software (Hamamatsu Photonics, Ltd., Shizuoka, Japan).

Steady-state excitation and emission spectra were recorded on a FluoTime 300 spectrometer from PicoQuant equipped with a 300 W ozone-free Xe lamp (250-900 nm), a 10 W Xe flash-lamp (250-900 nm, pulse width *ca*. 1 µs) with repetition rates of 0.1 – 300 Hz, double grating excitation monochromators (Czerny-Turner type, grating with 1200 lines/mm, blaze wavelength: 300 nm), diode lasers (pulse width < 80 ps) operated by a computer-controlled laser driver PDL-828 "Sepia II" (repetition rate up to 80 MHz, burst mode for slow and weak decays), two double-grating emission monochromators (Czerny-Turner, selectable gratings blazed at 500 nm with 2.7 nm/mm dispersion and 1200 lines/mm, or blazed at 1200 nm with 5.4 nm/mm dispersion and 600 lines/mm) with

adjustable slit width between 25 µm and 7 mm, Glan-Thompson polarizers for excitation (after the Xe-lamps) and emission (after the sample). Different sample holders (Peltier-cooled mounting unit ranging from -15 to 110 °C or an adjustable front-face sample holder), along with two detectors (namely a PMA Hybrid-07 from PicoQuant with transit time spread FWHM < 50 ps, 200 – 850 nm, or a H10330C-45-C3 NIR detector with transit time spread FWHM 0.4 ns, 950-1400 nm from Hamamatsu) were used. Steady-state spectra and photoluminescence lifetimes were recorded in TCSPC mode by a PicoHarp 300 (minimum base resolution 4 ps) or in MCS mode by a TimeHarp 260 (where up to several ms can be traced). Emission and excitation spectra were corrected for source intensity (lamp and grating) by standard correction curves. For samples with lifetimes in the ns order, an instrument response function calibration (IRF) was performed using a diluted Ludox® dispersion. Lifetime analysis was performed using the commercial EasyTau 2 software (PicoQuant). The quality of the fit was assessed by minimizing the reduced chi squared function (χ^2) and visual inspection of the weighted residuals and their autocorrelation. All solvents used were of spectrometric grade (Uvasol®, Merck).

1.3 Irradiation method (photoreactor)

Photoirradiation experiments were performed using a manufactured photoreactor and a high-power LED 365 nm, 55°, 3.6 V, 500 mA, by LG INNOTEK (λ_{LED} = 365 nm). The inside of the irradiation box is coated with aluminum to ensure total reflection of the light. To ensure consistent results, the distance between the sample and the light source (*d* = 7 cm) is maintained by fixing the cuvette in a stationary position throughout the entire irradiation process. For the kinetic measurements, the quartz (Hellma®) cuvette with a volume of 700 μL (outside dimensions 12.5 mm x 45 mm x 12.5 mm; layer thickness 10 x 2 mm) were completely filled and closed without any air inclusion. In contrast, for the cyclic measurements, the cuvette was filled with 400 μl of the corresponding sample (leaving a headspace with air).

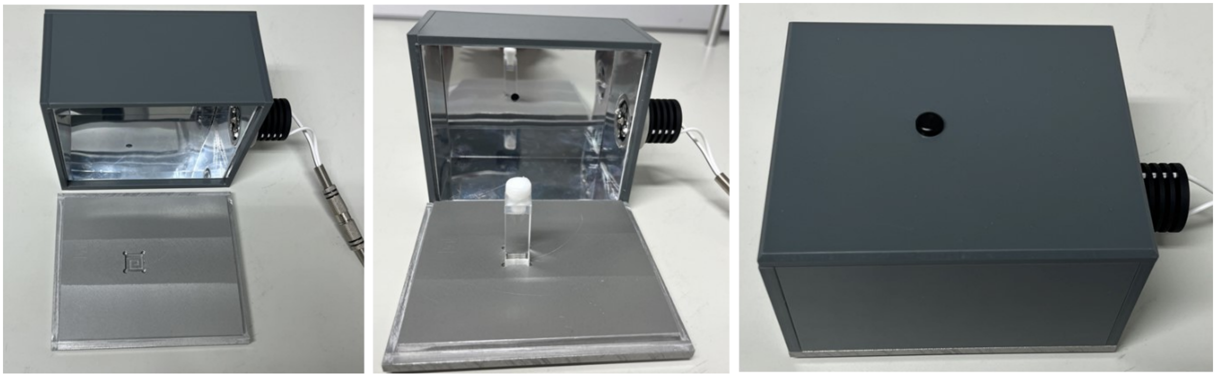

Figure S1: Pictures of the photoreactor.

2. Synthesis and characterization

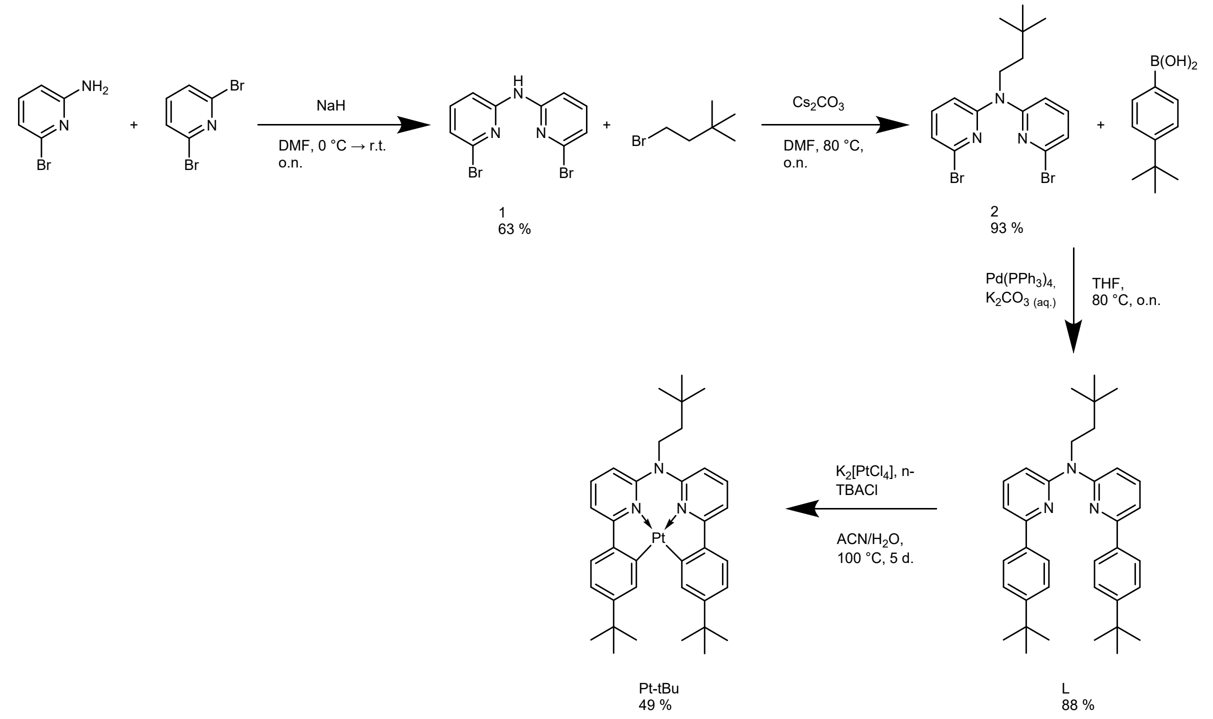

Figure S2: Synthetic route to obtain the target complex **Pt-*t*Bu**.

Compounds **1** and **2** have already been reported and were synthesized according to a modified procedure.[2,3]

2.1 Bis(6-bromopyridin-2-yl)-amine (1)

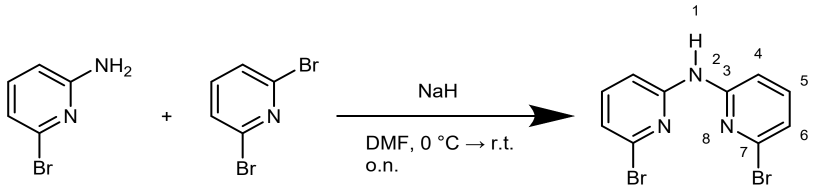

1

2-amino-6-bromopyridine (10.03 g, 57.97 mmol, 1.0 eq.) was dissolved in anhydrous DMF (80 mL) and the solution was cooled down to 0 °C. Under stirring, sodium hydride (4.31 g, 179.17 mmol, 3.1 eq.) was added. After 15 min of stirring, 2,6-dibromopyridine (13.71 g, 57.87 mmol, 1.0 eq.) was added to the solution. The reaction mixture was heated up to 60 °C and stirred overnight. After cooling to room temperature, distilled water was slowly added to the mixture to precipitate a white solid. The precipitate was filtrated and dried under reduced pressure. The crude product was purified by column chromatography (SiO_2, toluene). The product **1** was obtained as a white solid.

Yield: 11,97 g, 36.4 mmol, 63%.

EM-MS-ESI (MeOH, $C_{10}H_7N_3Br_2$): calcd. $[M+H]^+$ = 329.90593, found $[M+H]^+$ = 329.90617; calcd. $[M+Na]^+$ = 351.88787, found $[M+Na]^+$ = 351.88795.

^{1}H-NMR (400 MHz, CD_2Cl_2): δ (ppm) = 7.82 (s, 1H, H_1), 7.47 (m, 4H, $H_{4,5}$), 7.05 (m, 2H, H_6).

^{13}C{^{1}H}-NMR (101 MHz, CD_2Cl_2): δ (ppm) = 153.49 (C_3), 140.46 (C_5), 139.55 (C_7), 120.69 (C_6), 110.56 (C_4).

^{15}N-NMR (41 MHz, CD_2Cl_2): δ (ppm) = 116 (N_2), 275 (N_8).

2.2 Bis(6-bromopyridin-2-yl)-3,3-dimethylbutylamine (2)

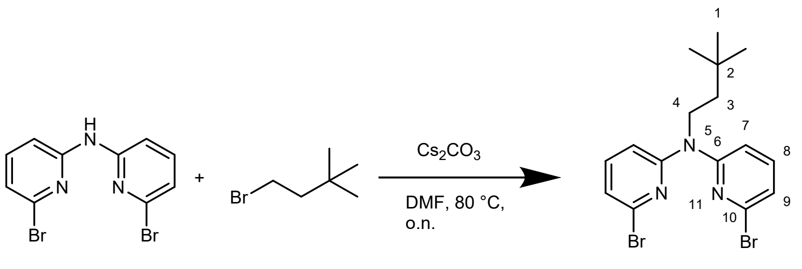

1 (2.00 g, 6.08 mmol, 1 eq.), 1-bromo-3,3-dimethylbutane (3.01 g, 2.62 mL, 18.24 mmol, 3 eq) and Cs_2CO_3 (11.88 g, 36.47 mmol, 6 eq.) were suspended in DMF (50 mL). The mixture was heated up to 80 °C and stirred overnight. The cooled reaction mixture was poured on distilled water (150 mL) and the aqueous phase was extracted with dichloromethane (5 x 30 mL). The combined organic phases were dried over anhydrous sodium sulfate and the solvent was removed under reduced pressure. The crude product was purified by column chromatography (SiO_2, CyH/EtOAc, 30:1). The product **2** was obtained as a white solid.

Yield: 2.33 g, 5.64 mmol, 93%.

EM-MS-ESI (MeOH, $C_{16}H_{19}N_3Br_2$): calcd. $[M+H]^+$ = 413.99987, found $[M+H]^+$ = 413.99986.

^{1}H-NMR (400 MHz, CD_2Cl_2): δ (ppm) 7.41 (d, $^3J_{HH}$ = 8.2, 7.7 Hz, 2H, H_8), 7.12 (dd, $^3J_{HH}$ = 8.2 Hz, 2H, H_7), 7.05 (d, $^3J_{HH}$ = 7.7 Hz, 2H, H_9), 4.15 (m, 2H, H_4), 1.58 (m, 2H, H_3), 1.00 (s, 9H, H_1).

^{13}C{^{1}H}-NMR (101 MHz, CD_2Cl_2): δ (ppm) = 156.69 (C_6), 139.99 (C_{10}), 139.81 (C_8), 121.09 (C_9), 113.12 (C_7), 45.84 (C_4), 41.03 (C_3), 30.24 (C_2), 29.44 (C_1).

^{15}N-NMR (41 MHz, CD_2Cl_2): δ (ppm) = 114 (N_5), 286 (N_{11}).

2.3 Bis-*N*-(6-(4-(*tert*-butyl)phenyl)pyridin-2-yl)-*N*-3,3-dimethylbutylamine (L)

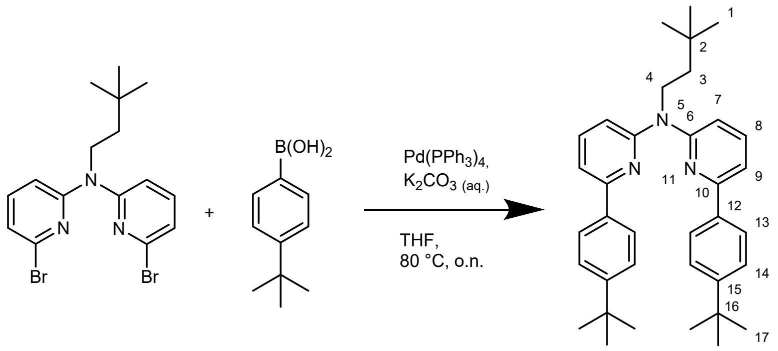

2 L

Bis(6-bromopyridin-2-yl)-3,3-dimethylbutylamine (250 mg, 0.61 mmol, 1 eq.), 4-*tert*-butylphenyl-boronic acid (323 mg, 1.83 mmol, 3 eq.) and $Pd(PPh_3)_4$ (70 mg, 0.06 mmol, 0.1 eq.) were dissolved in THF (20 mL) and a potassium carbonate solution (2 M, 3 mL) was added. The mixture was purged with argon for 15 min and stirred at 80 °C overnight. Distilled water (25 mL) was added to the mixture upon cooling, and the phases were separated. The aqueous phase was extracted with ethyl acetate (3 x 30 mL) and the combined organic phases were dried over anhydrous sodium sulfate. The solvent was removed under reduced pressure and the crude product was purified by column chromatography (SiO_2, CyH/EtOAc, 20:1 to 8:1). The product **L** was obtained as a yellow solid.

Yield: 277 mg, 0.53 mmol, 88%.

EM-MS-ESI (MeOH, $C_{36}H_{45}N_3$): calcd. $[M+H]^+$ = 520.36863, found $[M+H]^+$ = 520.36960.

^{1}H-NMR (500 MHz, CD_2Cl_2): δ (ppm) = 8.01 (m, 4H, H_{13}), 7.60 (t, $^3J_{HH}$ = 8.2, 7.6 Hz, 2H, H_8), 7.49 (m, 4H, H_{14}), 7.35 (d, $^3J_{HH}$ = 7.4 Hz, 2H, H_9), 7.15 (d, $^3J_{HH}$ = 8.2 Hz, 2H, H_7), 4.47 (m, 2H, H_4), 1.78 (m, 2H, H_3), 1.37 (s, 18H, H_{17}), 1.07 (s, 9H, H_1).

^{13}C{^{1}H}-NMR (126 MHz, CD_2Cl_2): δ (ppm) = 157.26 (C_6), 155.74(C_{10}), 152.40 (C_{15}), 137.99 (C_8), 137.01 (C_{12}), 126.69 (C_{13}), 125.91 (C_{14}), 113.09 (C_7), 112.85 (C_9), 45.12 (C_4), 41.53 (C_3), 34.98 (C_{16}), 31.48 (C_{17}), 30.27 (C_2), 29.75 (C_1).

^{15}N-NMR (51 MHz, CD_2Cl_2): δ (ppm) = 278 (N_{11}), 113 (N_5).

2.4 (κ^4_{CNNC}-bis-*N*-(6-(4-(*tert*-butyl)phenylide)pyridin-2-yl)-*N*-3,3-dimethylbutylamine)-platinum (II) (Pt-*t*Bu)

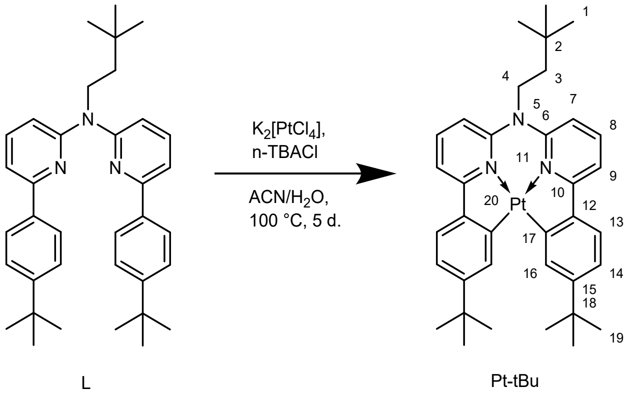

L (250 mg, 0.48 mmol, 1 eq.), $K_2[PtCl_4]$ (240 mg, 0.58 mmol, 1.2 eq.) and tetrabutylammonium chloride (catalytic amount) were suspended in a mixture of ACN/H_2O (10:5 mL). The reaction mixture was purged with argon for 15 min and stirred at 100 °C for 5 days. After that, the solvent was evaporated under reduced pressure and the crude product was purified by column chromatography (SiO_2, CyH/acetone, 15:1 to 8:1). The product **Pt-*t*Bu** was obtained as a yellow solid.

Yield: 168 mg, 0.24 mmol, 49%.

EM-MS-ESI (MeOH, $C_{36}H_{43}N_3Pt$): calcd. $[M+H]^+$ = 713.31806, found $[M+H]^+$ = 713.31871; calcd. $[M+Na]^+$ = 735.30001, found $[M+Na]^+$ = 735.30068.

^{1}H-NMR (400 MHz, $CDCl_3$): δ (ppm) 8.40 (d, $^4J_{HH}$ = 1.9 Hz, $^3J_{HPt}$ = 54 Hz, 2H, H_{16}), 7.82 (t, $^3J_{HH}$ = 8.0 Hz, 2H, H_8), 7.64 (d, $^3J_{HH}$ = 8.2 Hz, 2H, H_{13}), 7.50 (d, $^3J_{HH}$ = 8.0 Hz, 2H, H_9), 7.21 (dd, $^3J_{HH}$ = 8.2 Hz, $^4J_{HH}$ = 1.9 Hz, 2H, H_{14}), 7.11 (d, $^3J_{HH}$ = 8.1 Hz, 2H, H_7), 4.19 (m, 2H, H_4), 1.54 (m, 2H, H_3), 1.47 (s, 18H, H_{19}), 0.86 (s, 9H, H_{11}).

^{13}C{^{1}H}-NMR (101 MHz, $CDCl_3$): δ (ppm) = 164.47 ($^3J_{CPt}$ = 65 Hz, C_{10}), 152.18 ($^3J_{CPt}$ = 64 Hz, C_{15}), 150.70 (C_6), 148.35 ($^1J_{CPt}$ = 1111 Hz, C_{17}), 144.88 (C_{12}), 137.38 (C_8), 133.03 ($^2J_{CPt}$ = 99 Hz, C_{16}), 123.65 ($^3J_{CPt}$ = 42 Hz, C_{13}), 120.47 (C_{14}), 112.94 (C_7), 112.04 (C_9), 52.19 (C_4), 41.52 (C_3), 35.12 (C_{18}), 31.70 (C_{19}), 30.02 (C_2), 29.25 (C_1).

^{15}N-NMR (41 MHz, $CDCl_3$): δ (ppm) = 109 (N_5), 227 (N_{11}).

^{195}Pt-NMR (86 MHz, $CDCl_3$): δ (ppm) = -3514.

3. NMR and mass spectra

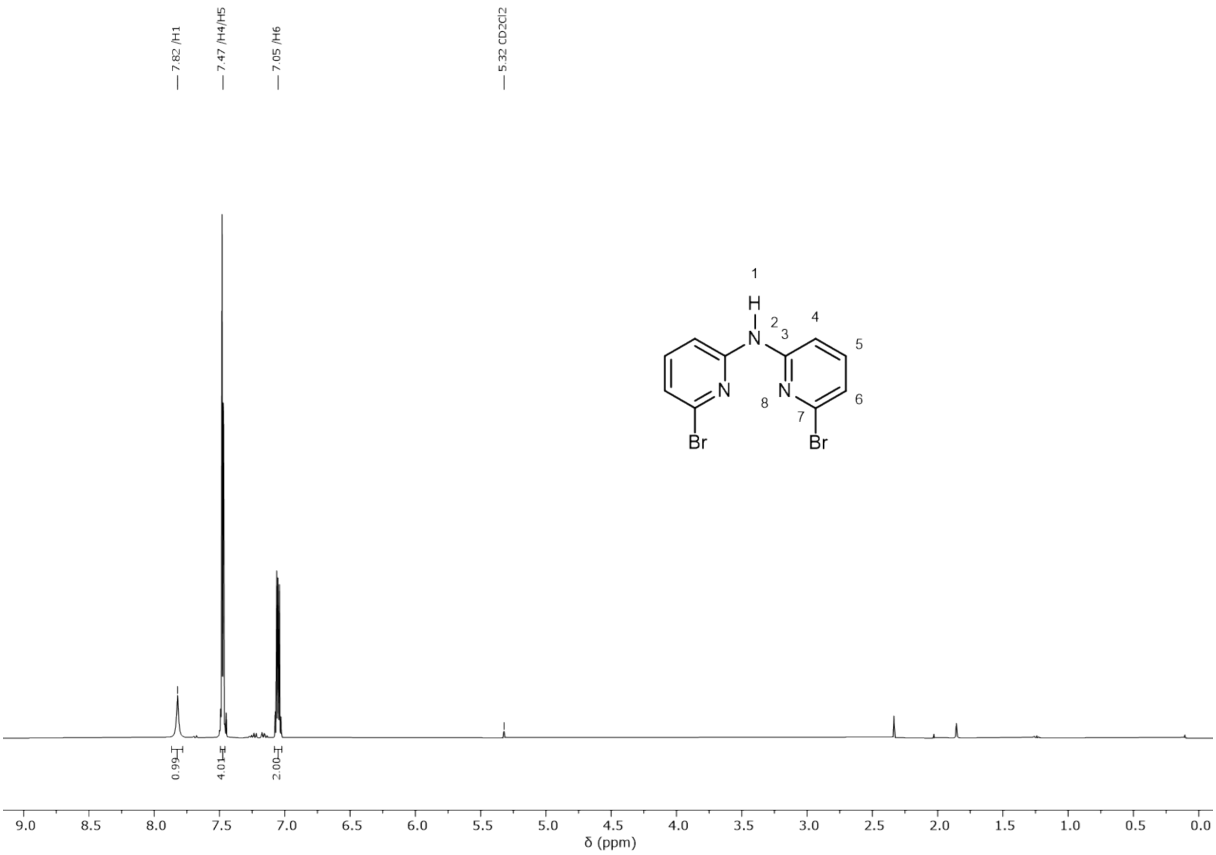

Figure S3: ^{1}H-NMR spectrum (400 MHz, CD_2Cl_2) of **1**.

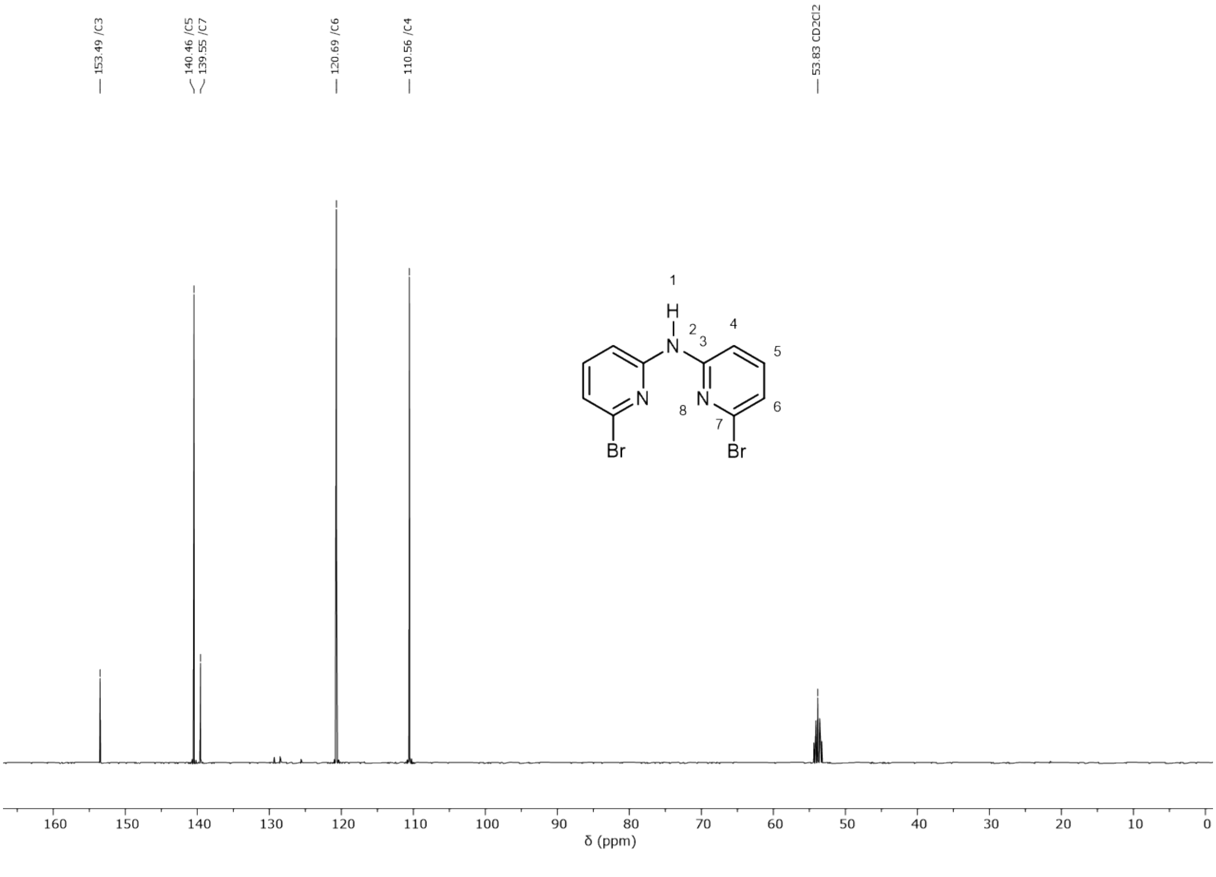

Figure S4: ^{13}C-NMR spectrum (101 MHz, CD_2Cl_2) of **1**.

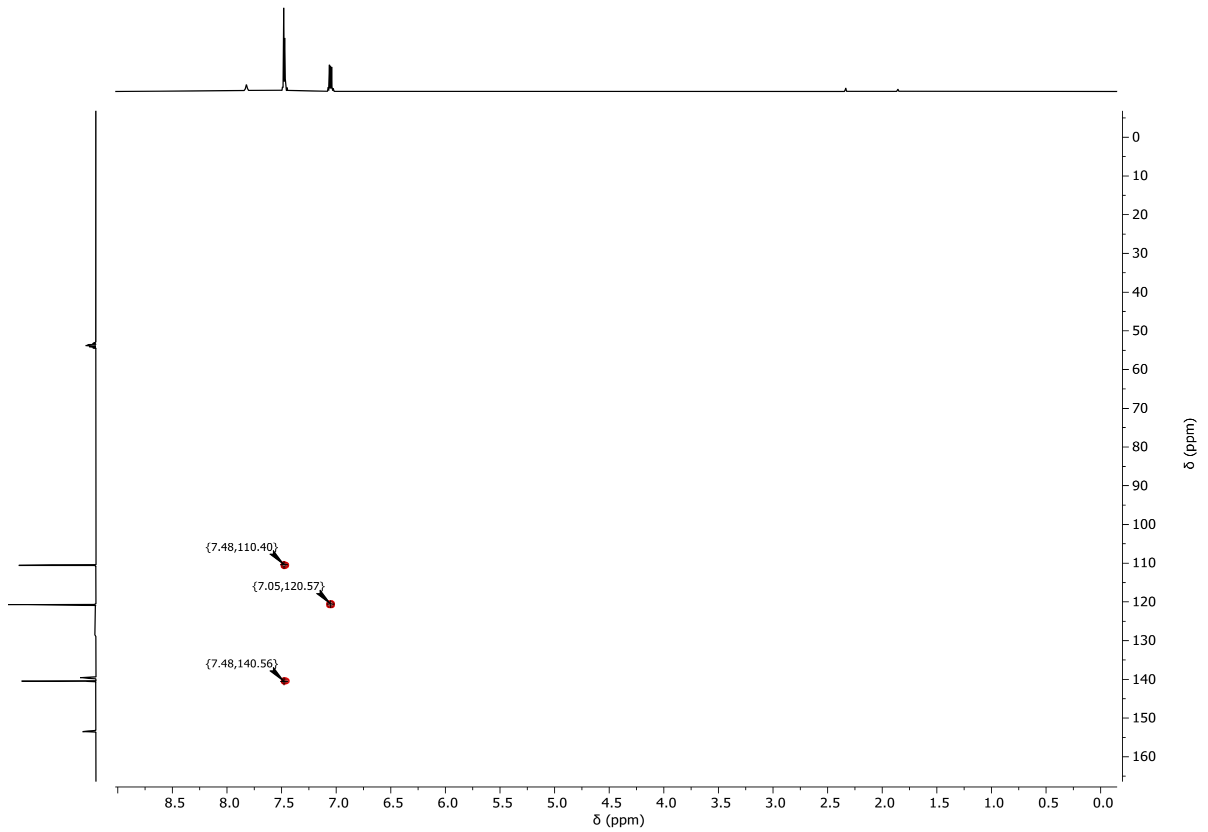

Figure S5: ^{1}H, ^{13}C-HSQC spectrum (400 MHz, 101 MHz, CD_2Cl_2) of **1**.

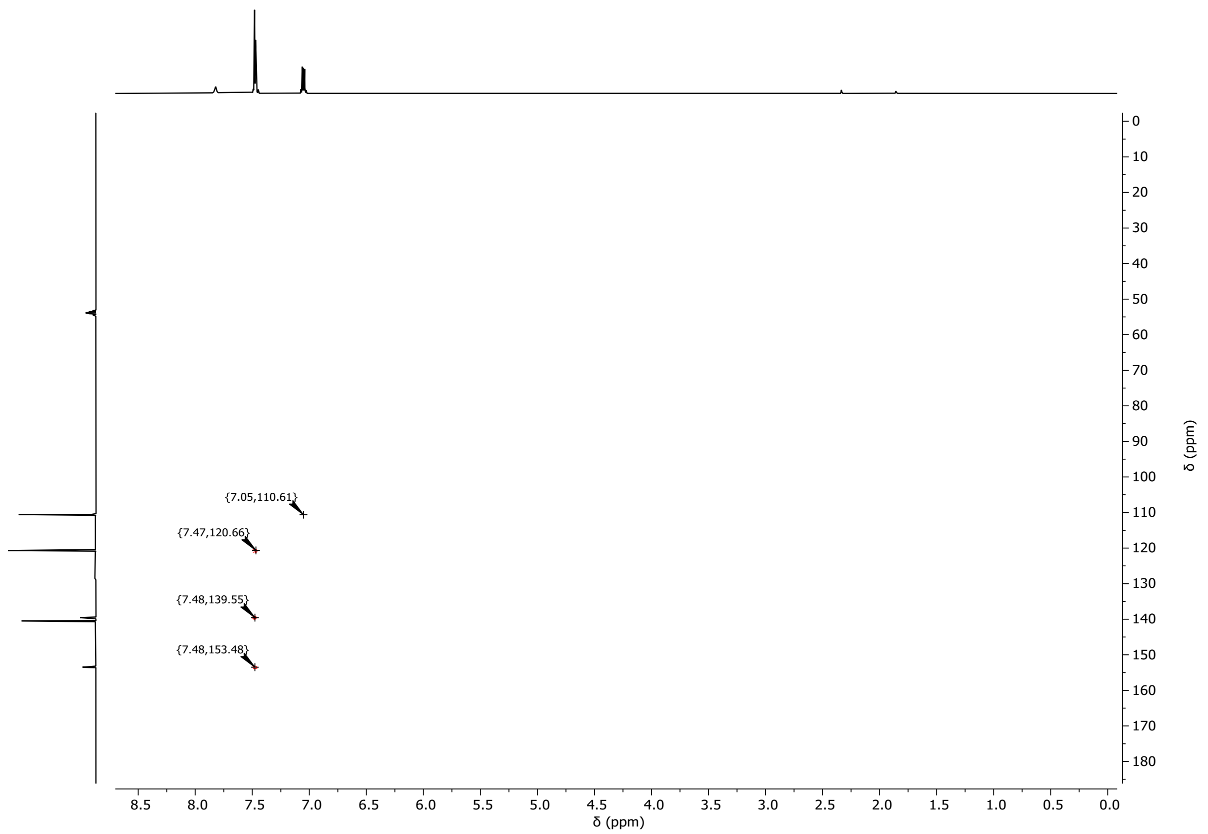

Figure S6: ^{1}H, ^{13}C-HMBC spectrum (400 MHz, 101 MHz, CD_2Cl_2) of **1**.

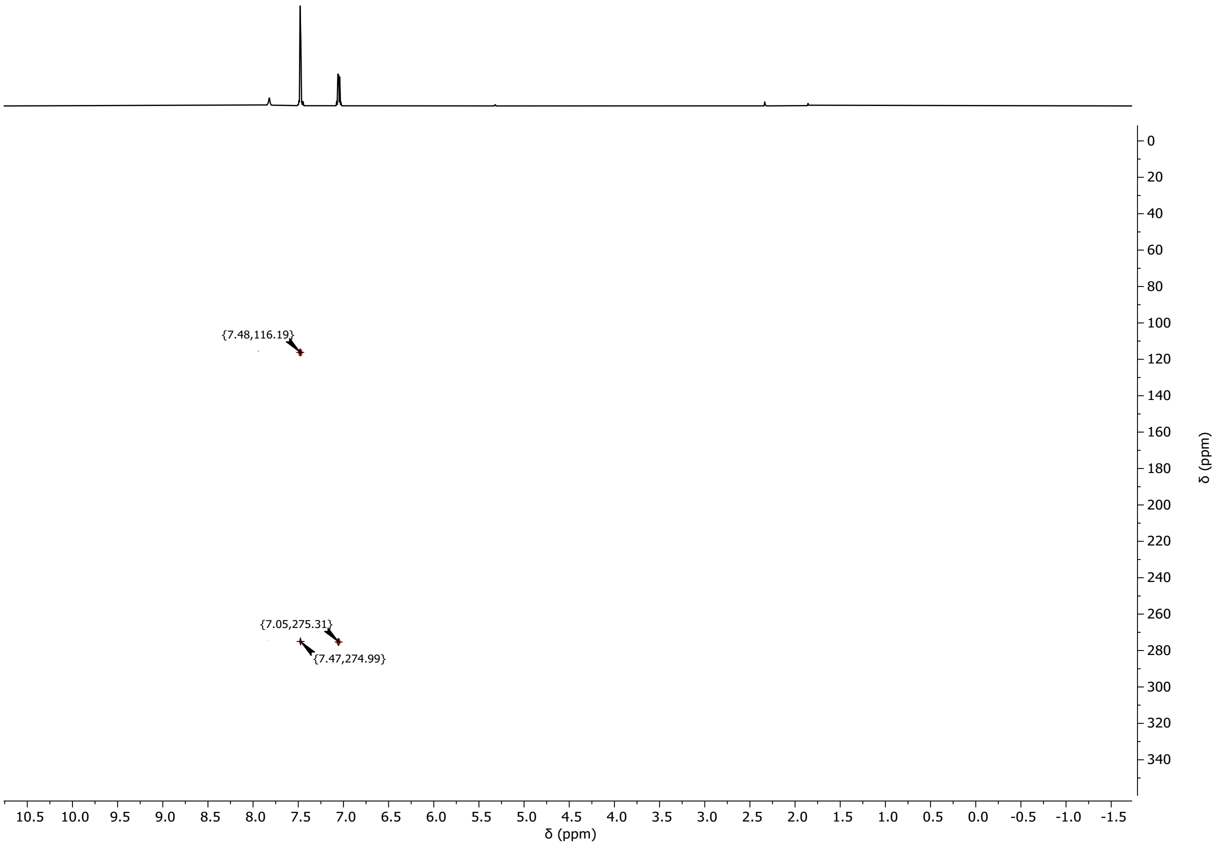

Figure S7: ^{1}H, ^{15}N-HMBC spectrum (400 MHz, 41 MHz, CD_2Cl_2) of **1**.

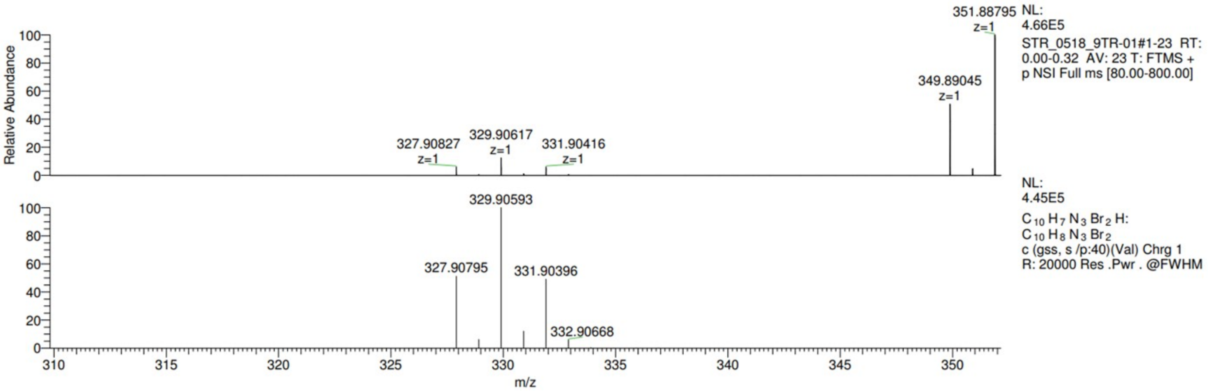

Figure S8: Mass spectrum of **1** (top). Additional simulation of the $[\mathbf{1}+H]^{+}$ adduct (bottom).

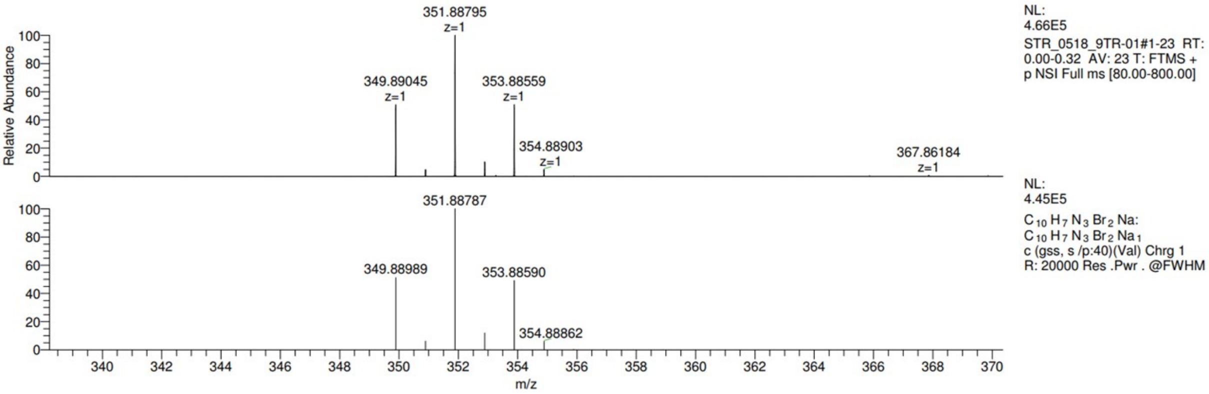

Figure S9: Mass spectrum of **1** (top). Additional simulation of the $[\mathbf{1}+Na]^{+}$ adduct (bottom).

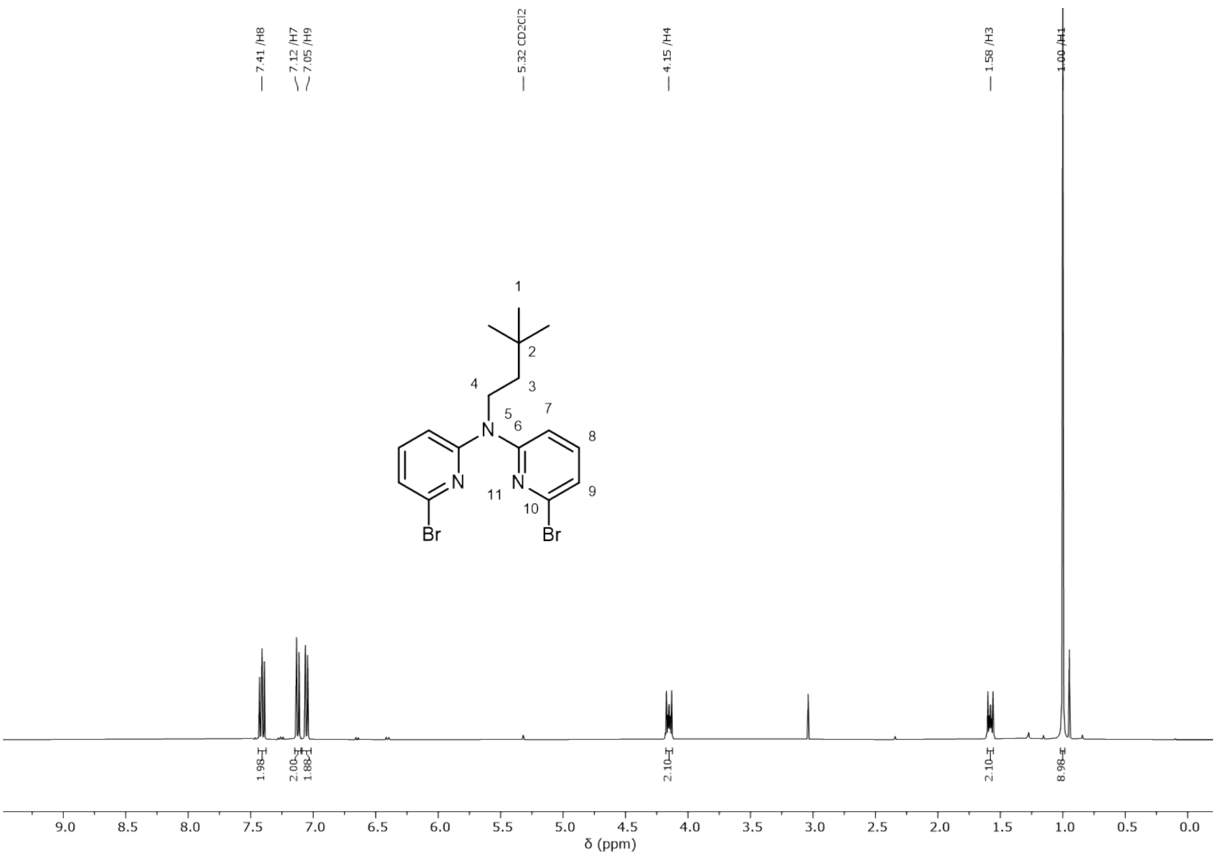

Figure S10: ^{1}H-NMR spectrum (400 MHz, CD_2Cl_2) of **2**.

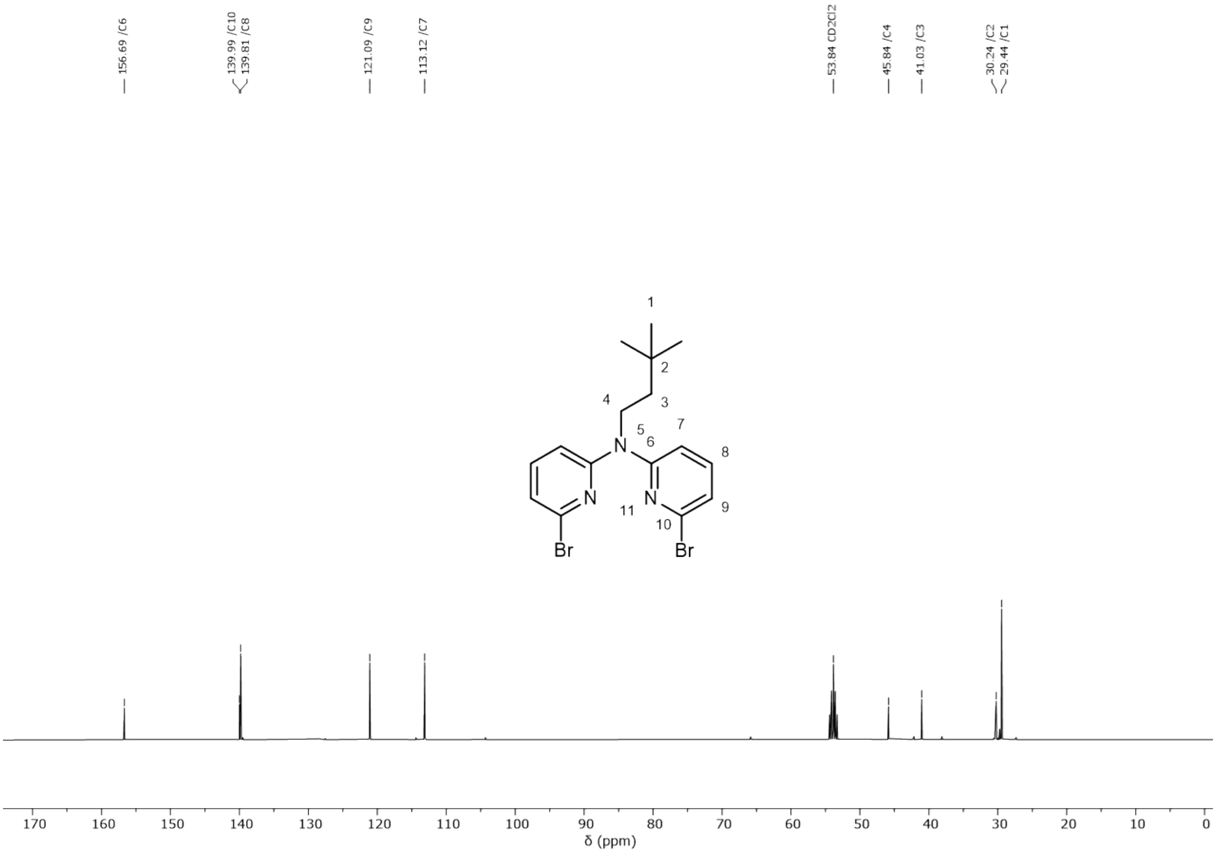

Figure S11: ^{13}C-NMR spectrum (101 MHz, CD_2Cl_2) of **2**.

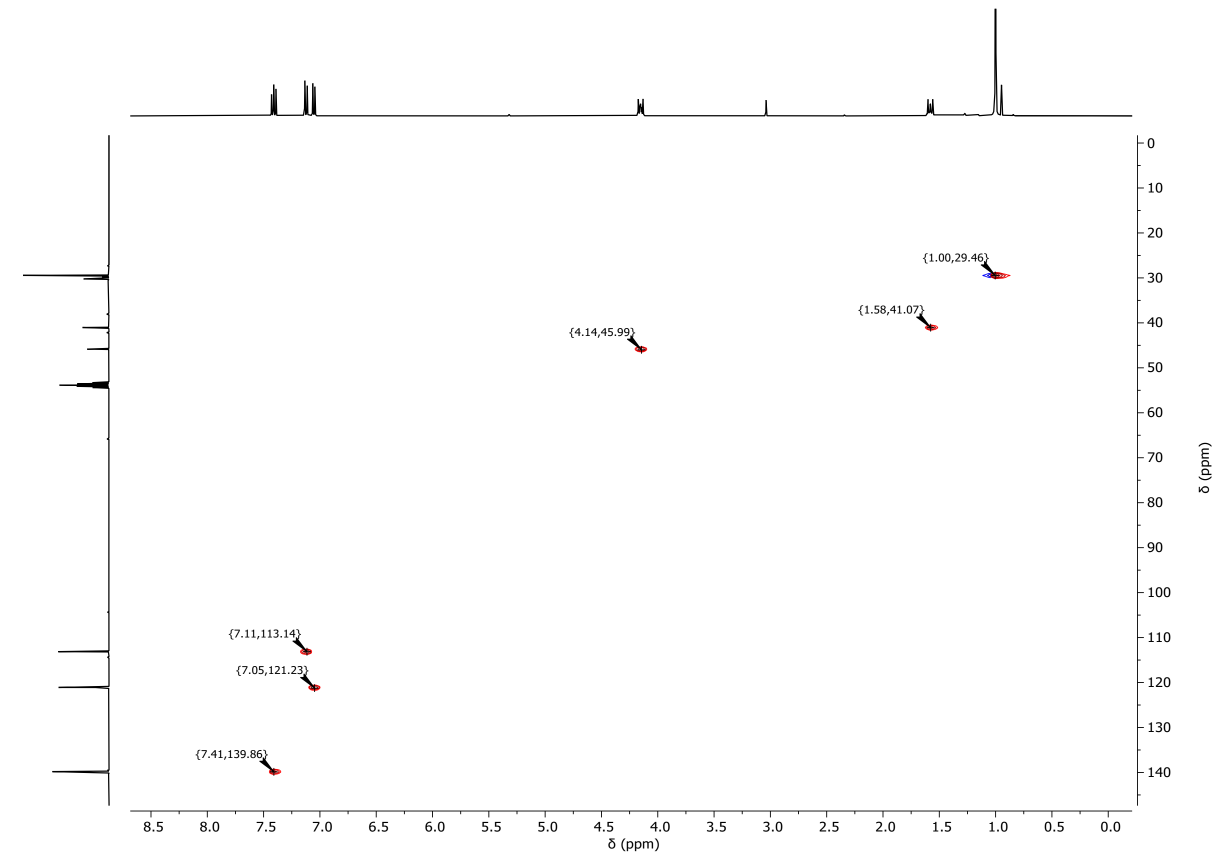

Figure S12: ^{1}H, ^{13}C-HSQC spectrum (400 MHz, 101 MHz, CD_2Cl_2) of **2**.

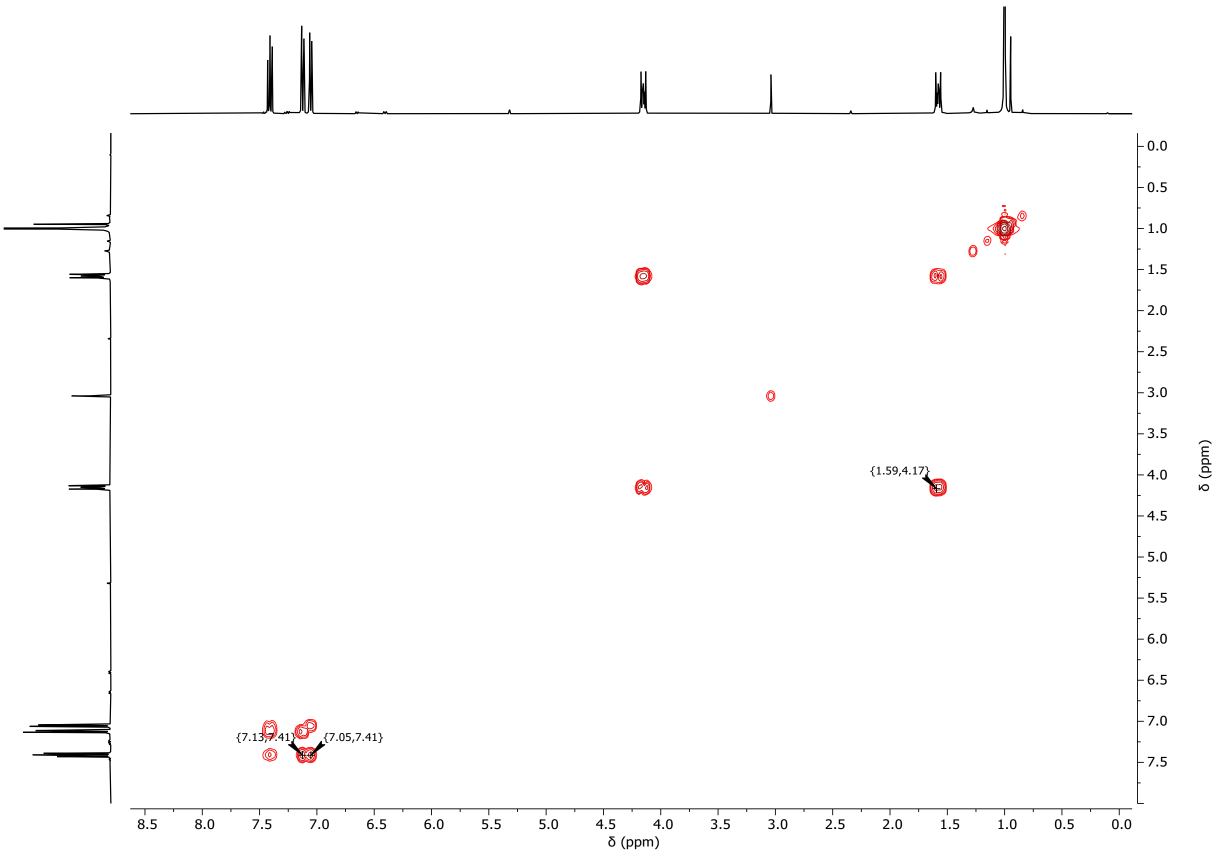

Figure S13: ^{1}H, ^{1}H-COSY spectrum (400 MHz, CD_2Cl_2) of **2**.

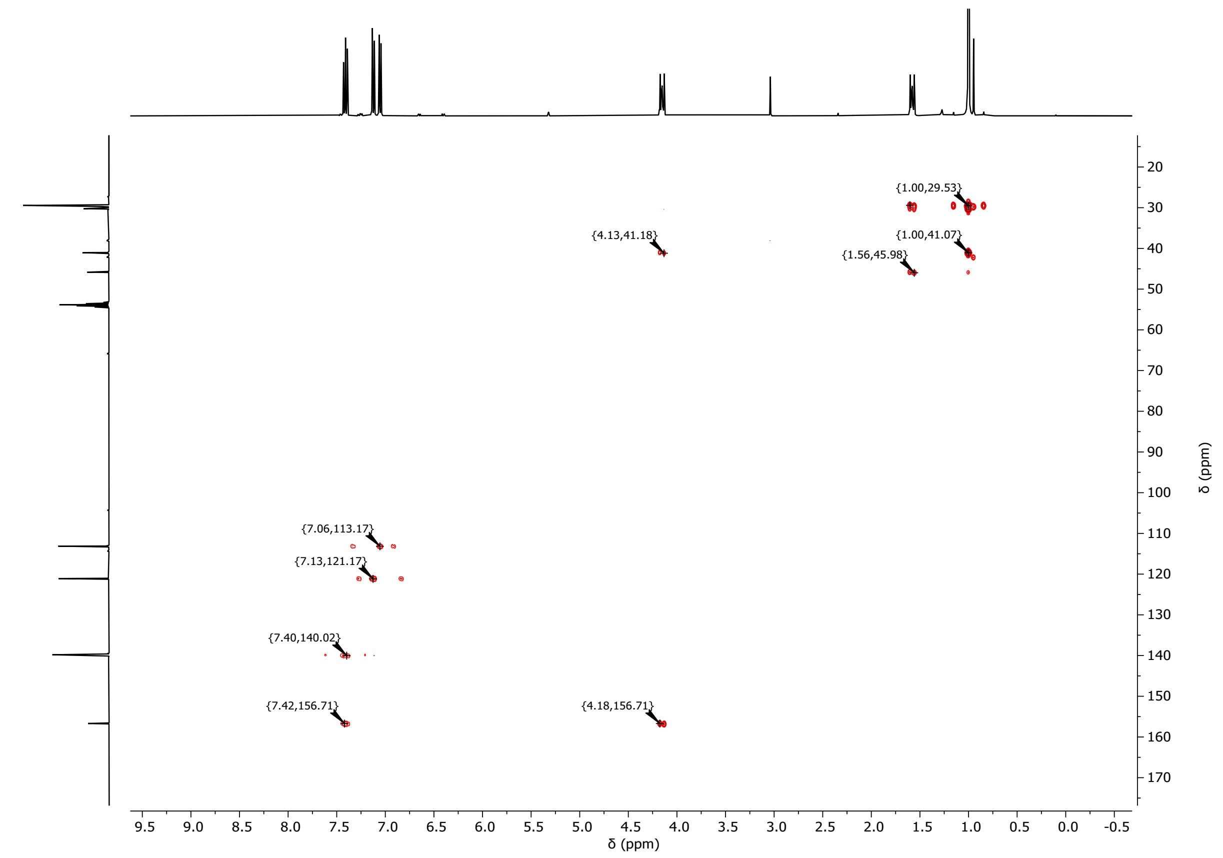

Figure S14: ^{1}H, ^{13}C-HMBC spectrum (400 MHz, 101 MHz, CD_2Cl_2) of **2**.

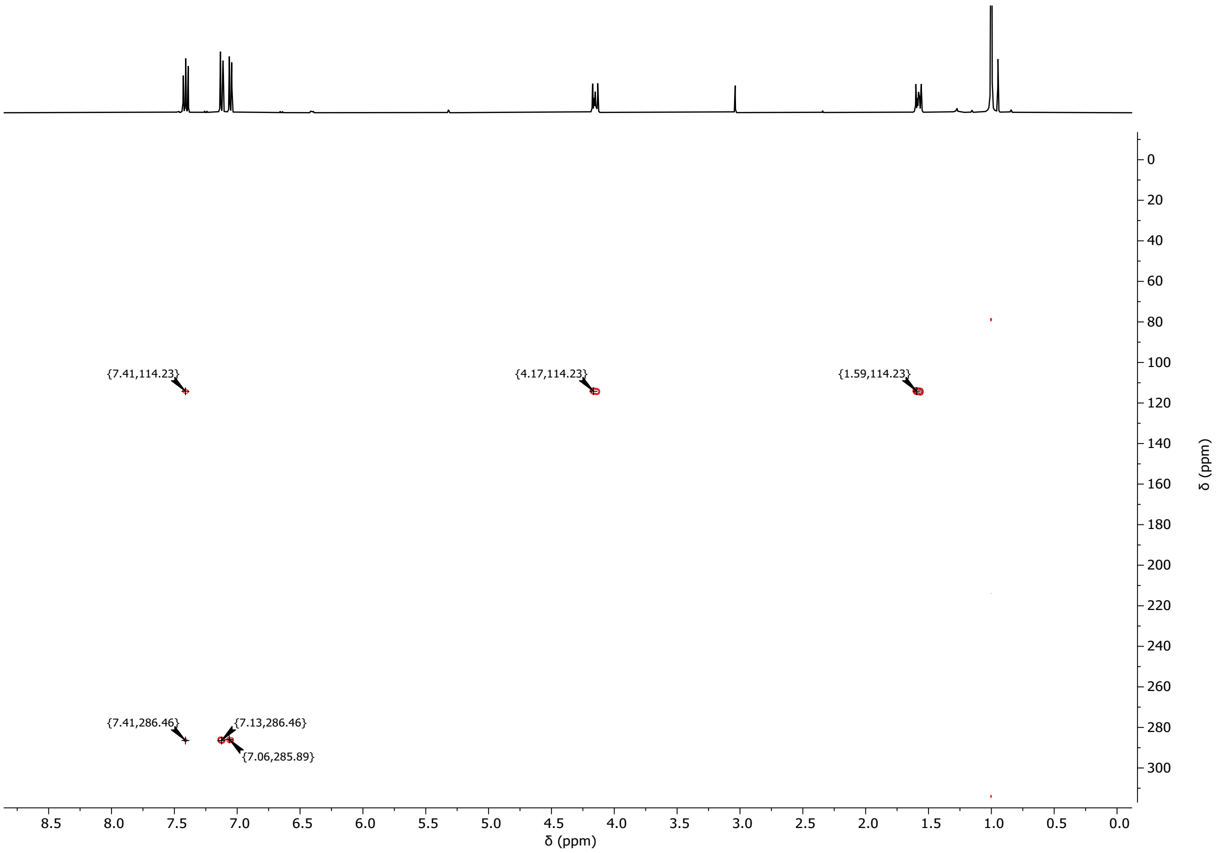

Figure S15: ^{1}H, ^{15}N-HMBC spectrum (400 MHz, 41 MHz, CD_2Cl_2) of **2**.

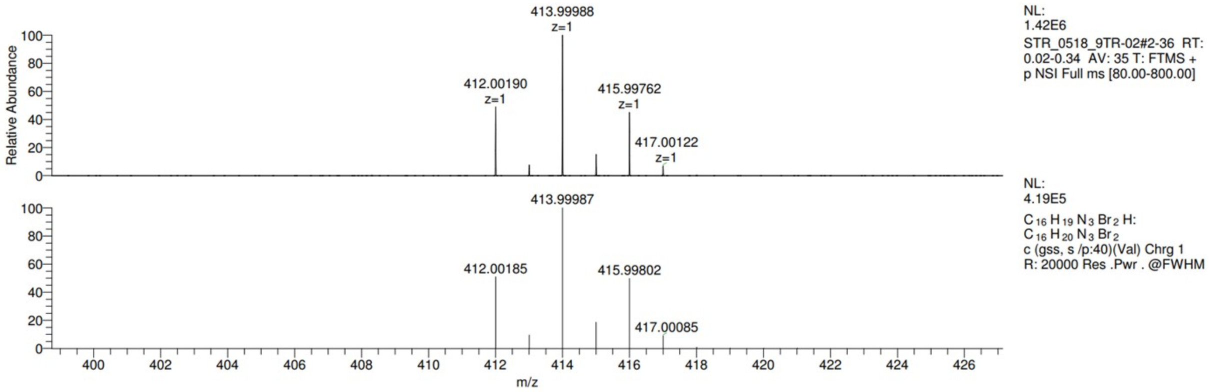

Figure S16: Mass spectrum of **2** (top). Additional simulation of the [**2**+H]$^{+}$ adduct (bottom).

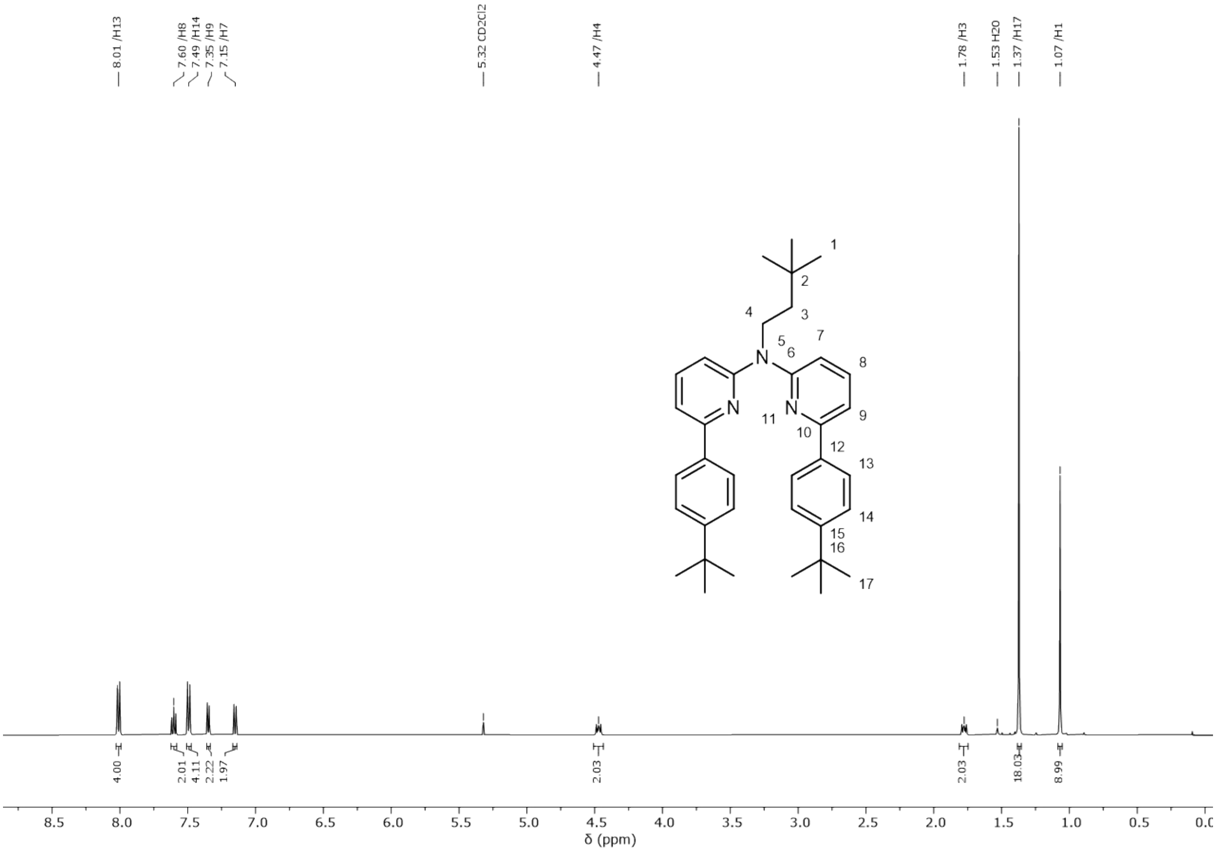

Figure S17: ^{1}H-NMR spectrum (500 MHz, CD_2Cl_2) of **L**.

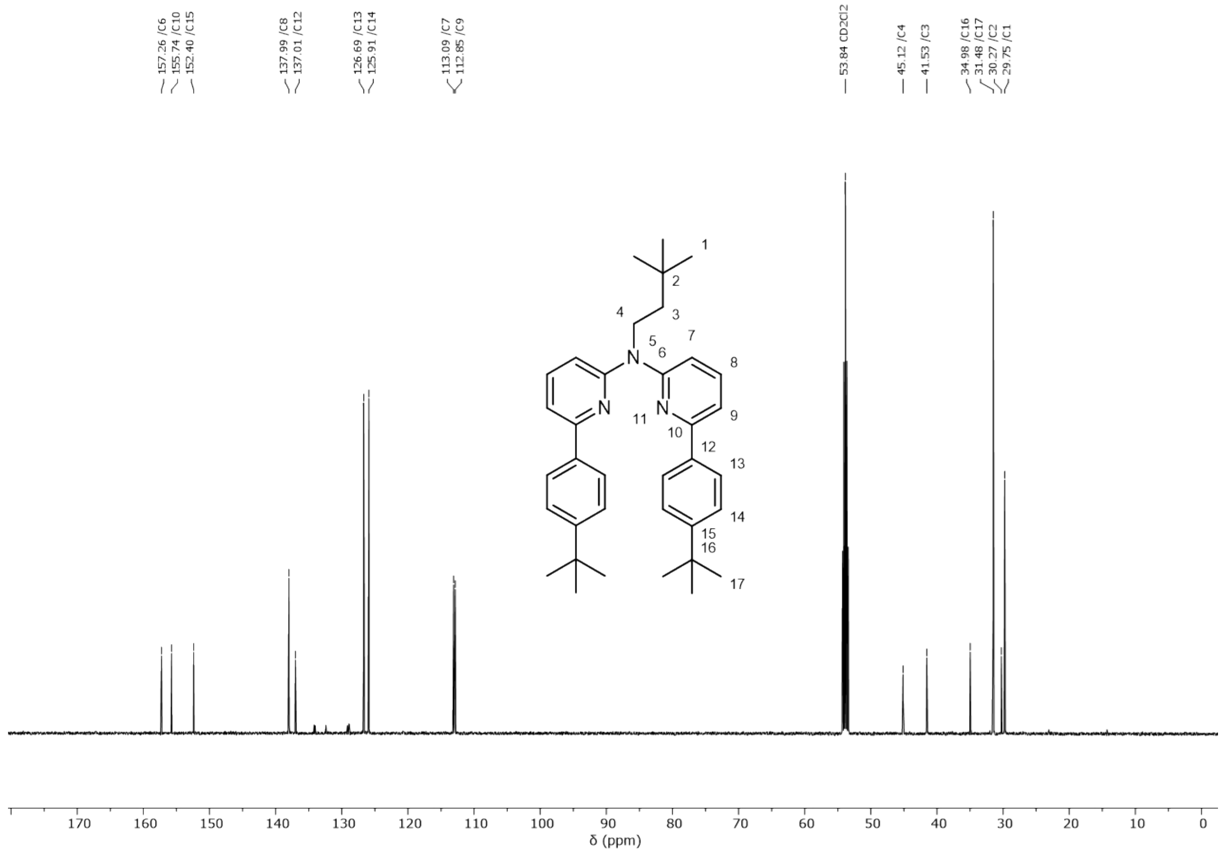

Figure S18: ^{13}C-NMR spectrum (126 MHz, CD_2Cl_2) of **L**.

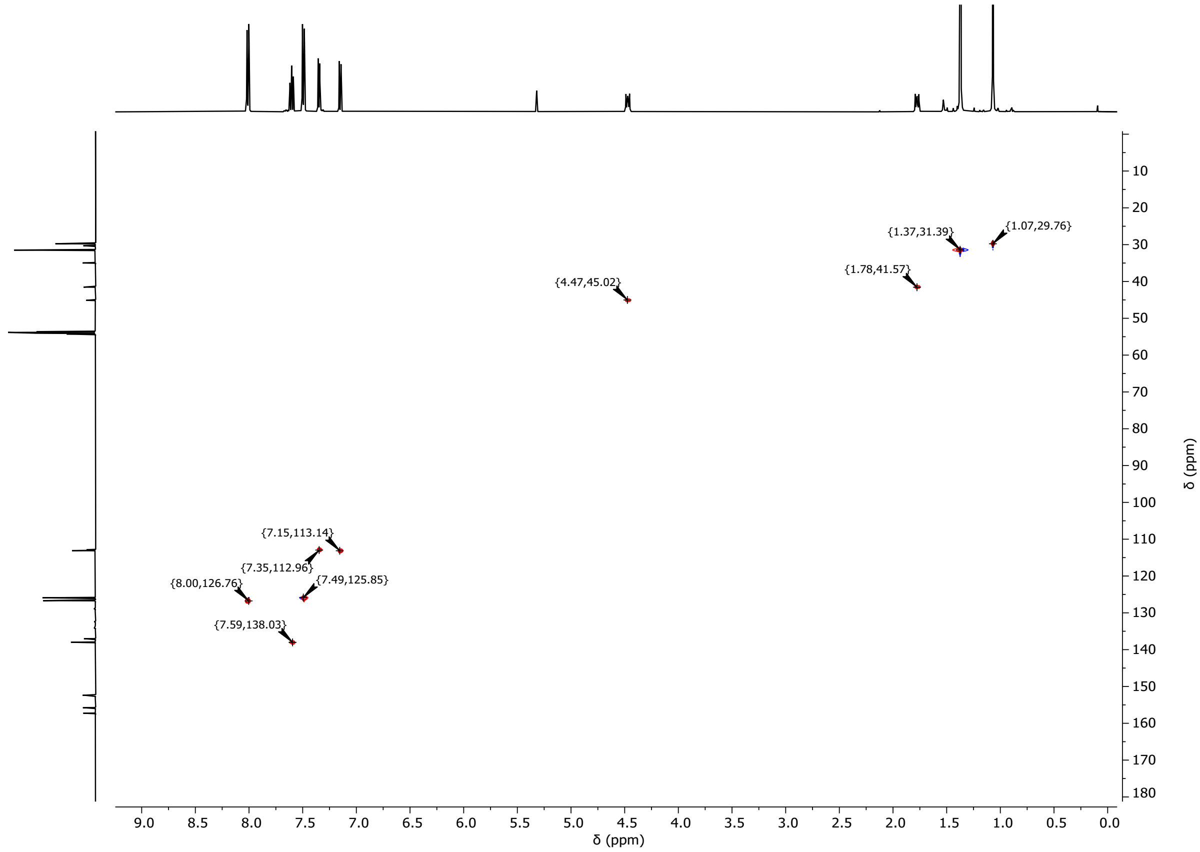

Figure S19: ^{1}H, ^{13}C-HSQC spectrum (500 MHz, 126 MHz, CD_2Cl_2) of **L**.

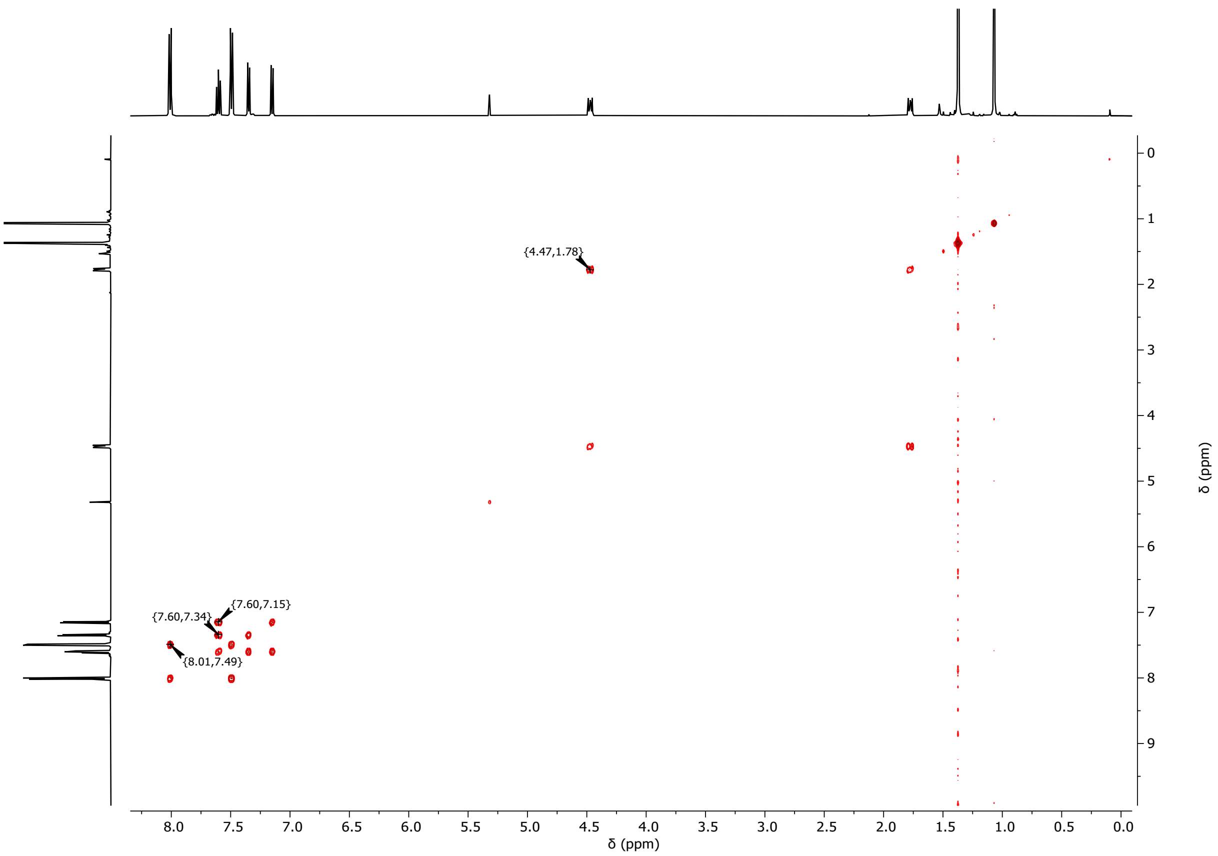

Figure S20: ^{1}H, ^{1}H-COSY spectrum (500 MHz, CD_2Cl_2) of **L**.

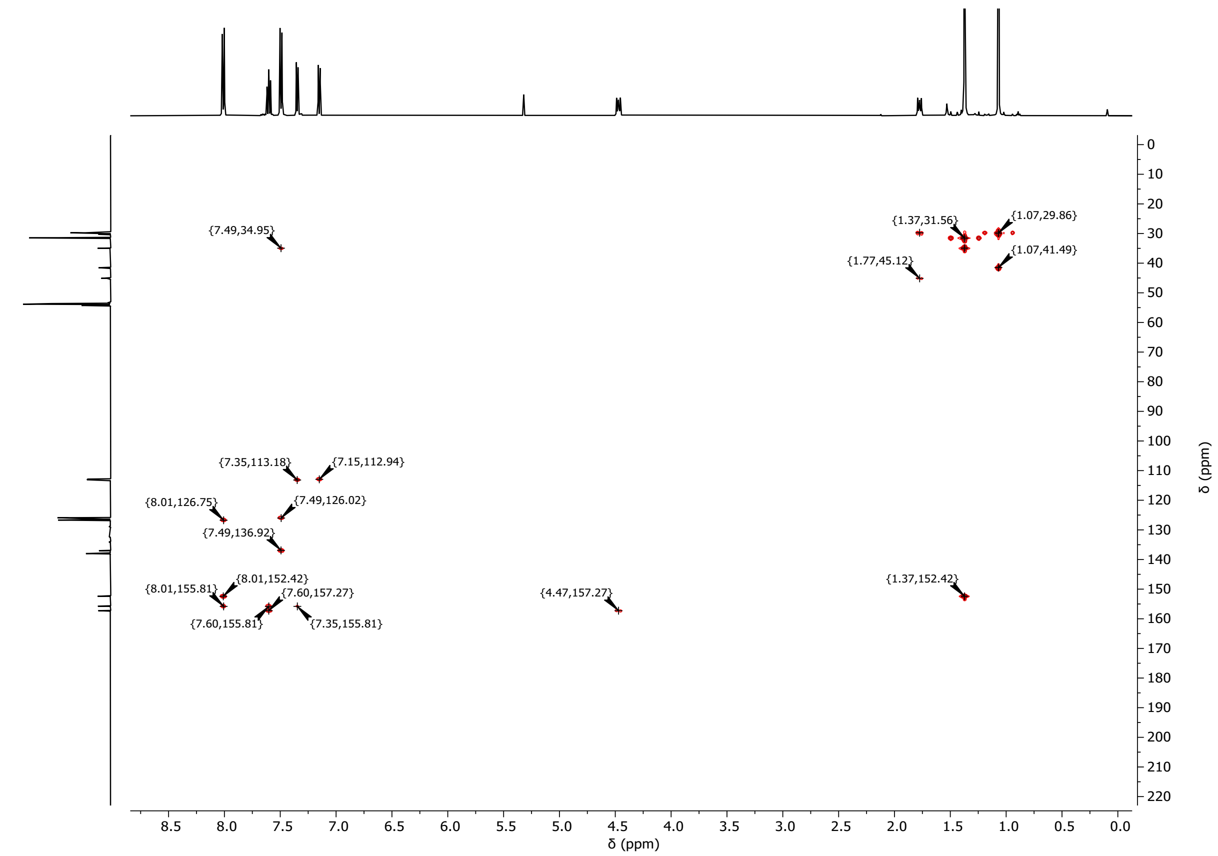

Figure S21: ^{1}H, ^{13}C-HMBC spectrum (500 MHz, 126 MHz, CD_2Cl_2) of **L**.

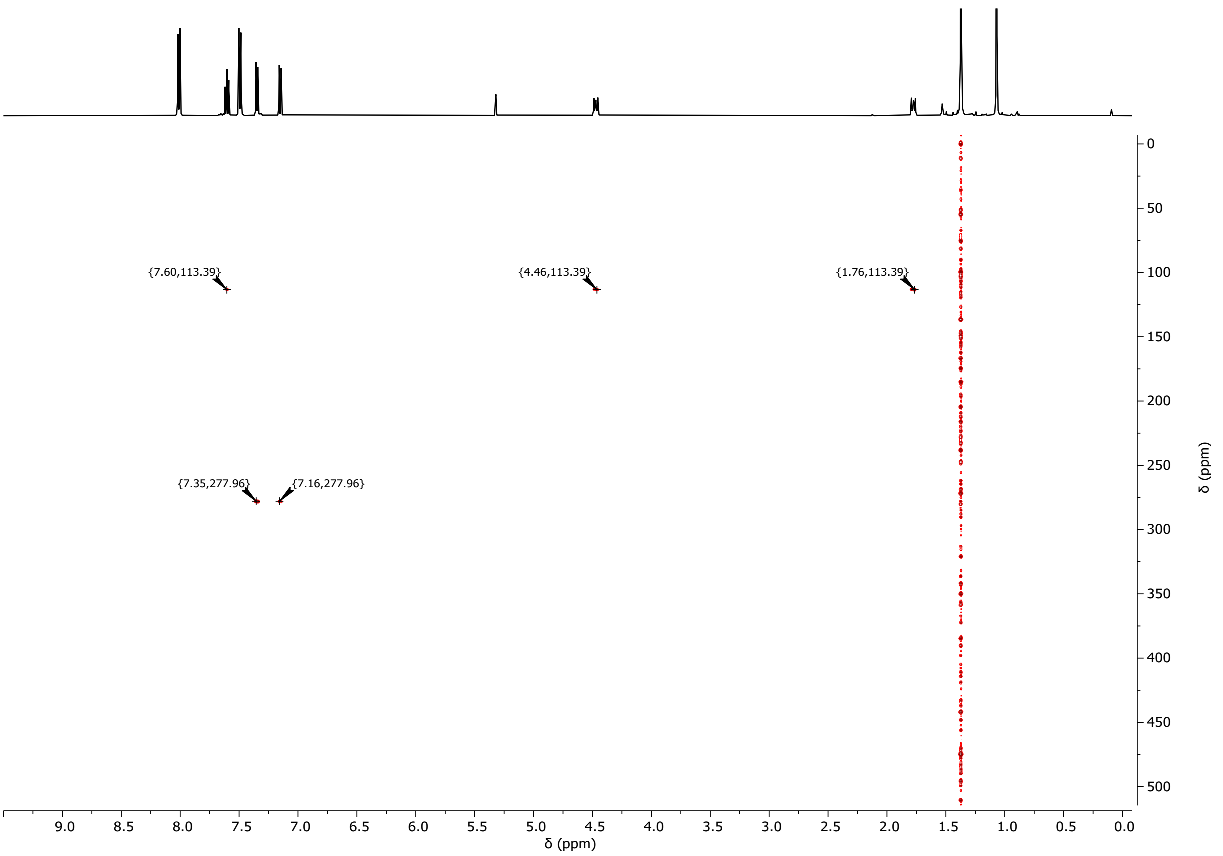

Figure S22: ^{1}H, ^{15}N-HMBC spectrum (500 MHz, 51 MHz, CD_2Cl_2) of **L**.

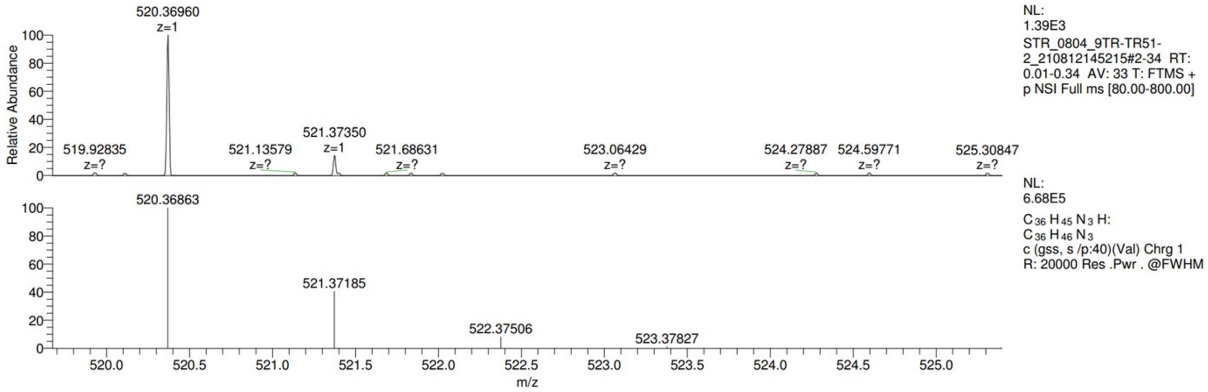

Figure S23: Mass spectrum of **L** (top). Additional simulation of the $[\mathbf{L}+H]^+$ adduct (bottom).

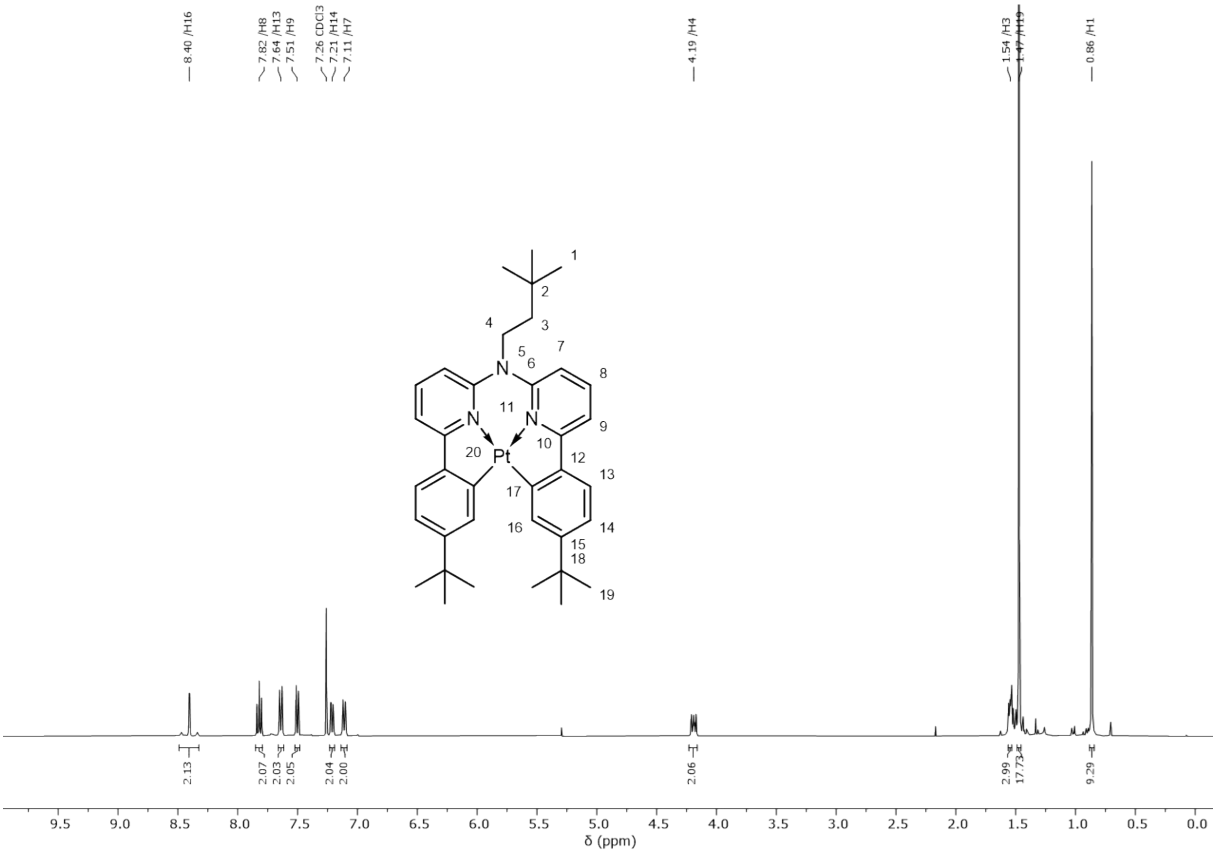

Figure S24: ^{1}H-NMR spectrum (400 MHz, $CDCl_3$) of **Pt-*t*Bu**.

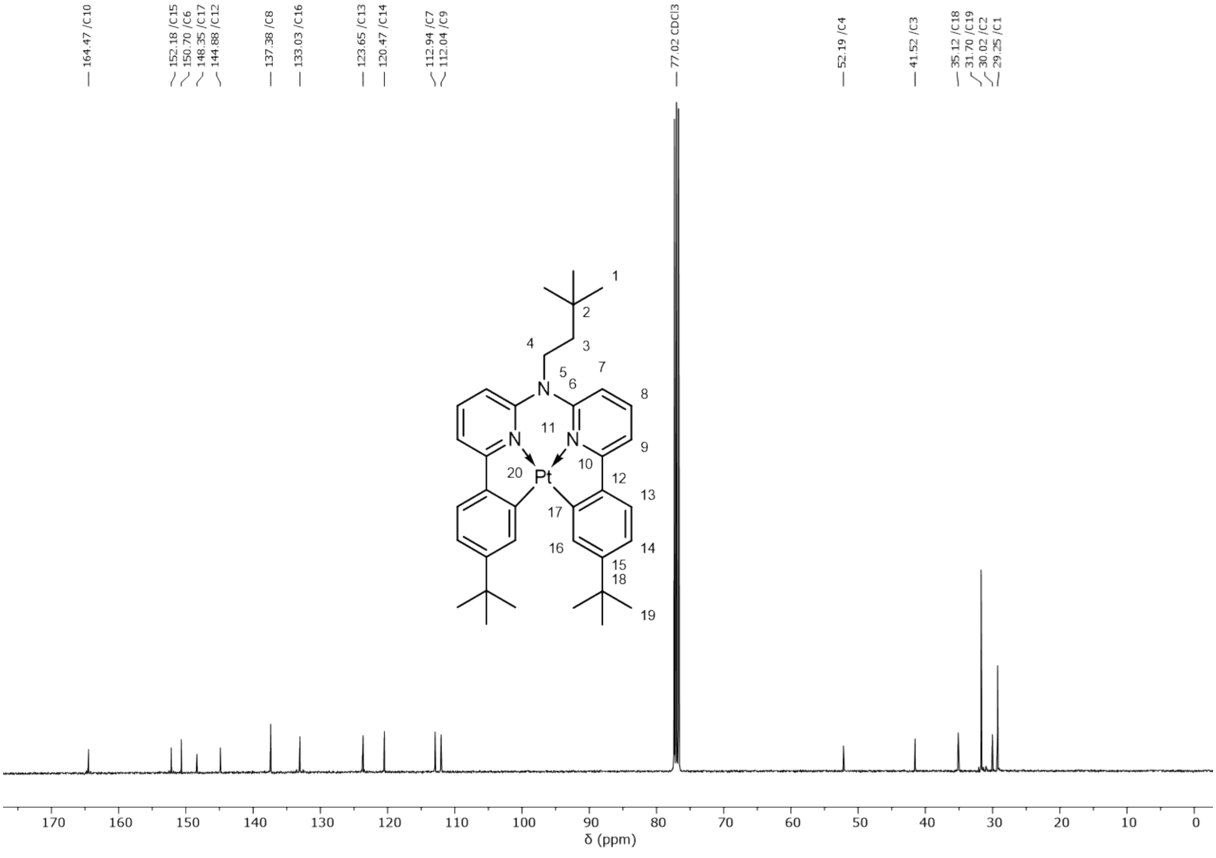

Figure S25: ^{13}C-NMR spectrum (101 MHz, $CDCl_3$) of **Pt-*t*Bu**.

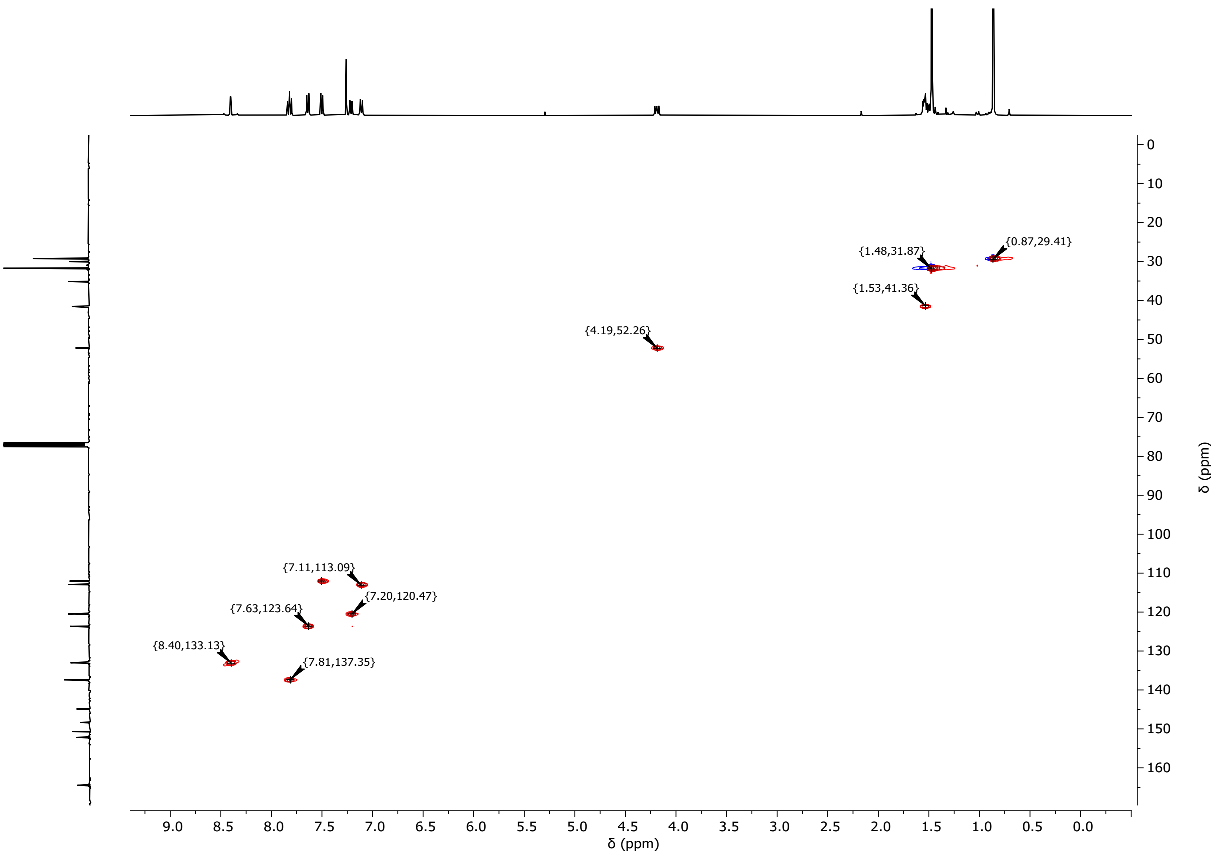

Figure S26: ^{1}H, ^{13}C-HSQC spectrum (400 MHz, 101 MHz, $CDCl_3$) of **Pt-*t*Bu**.

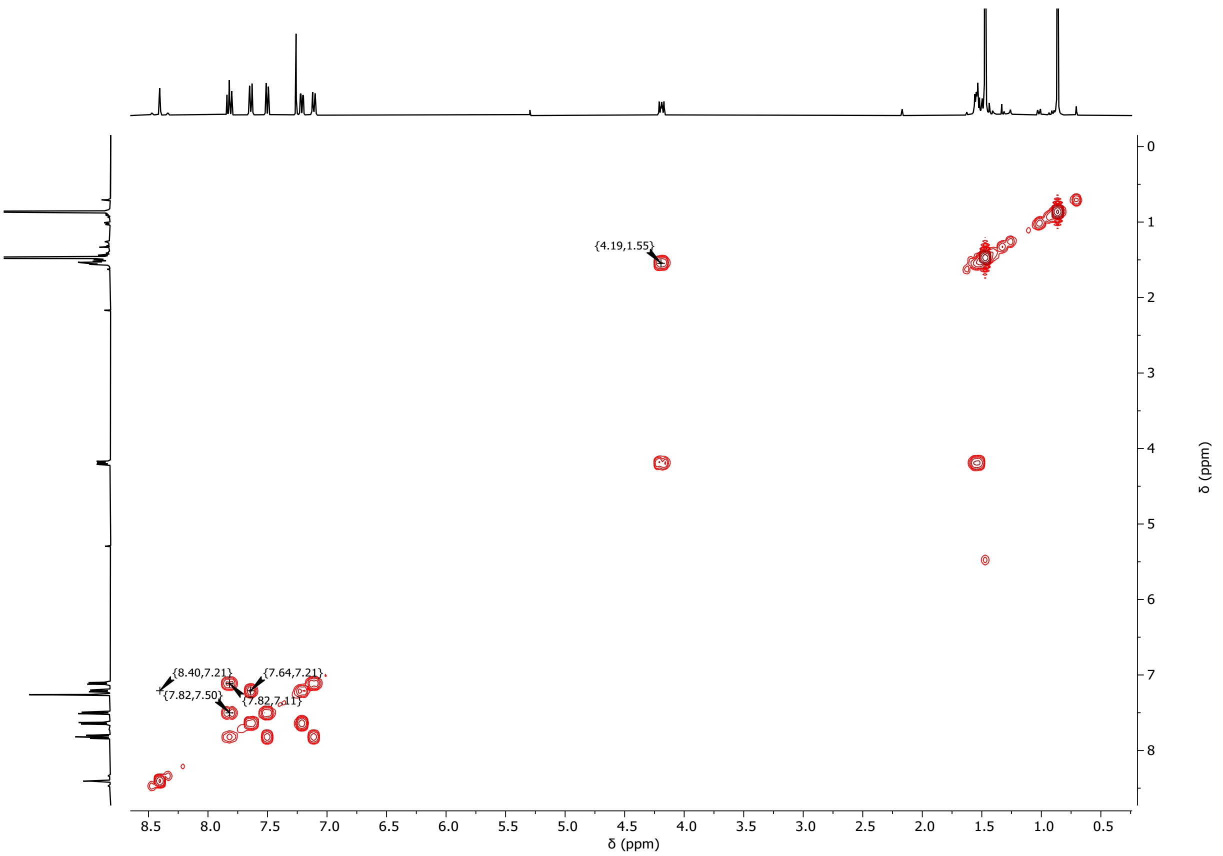

Figure S27: ^{1}H, ^{1}H-COSY spectrum (400 MHz, $CDCl_3$) of **Pt-*t*Bu**.

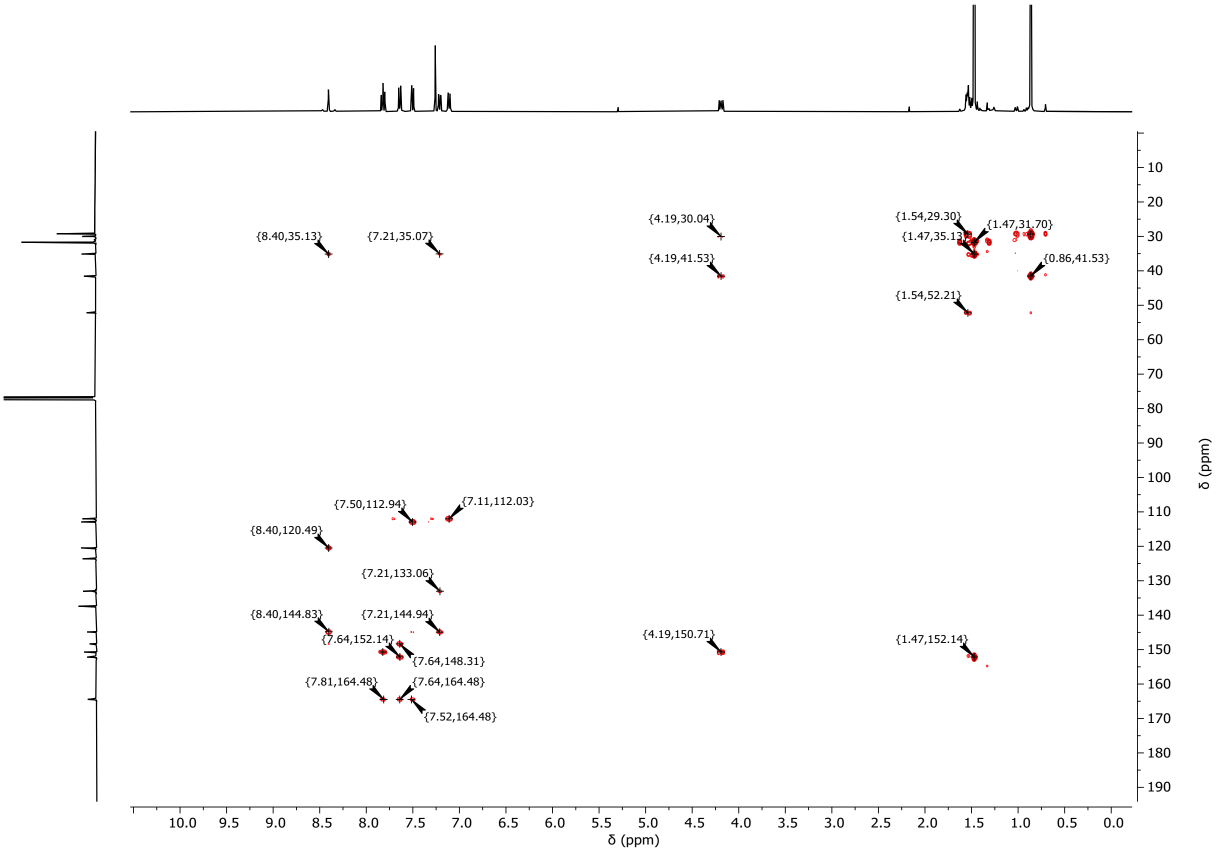

Figure S28: ^{1}H, ^{13}C-HMBC spectrum (400 MHz, 101 MHz, $CDCl_3$) of **Pt-*t*Bu**.

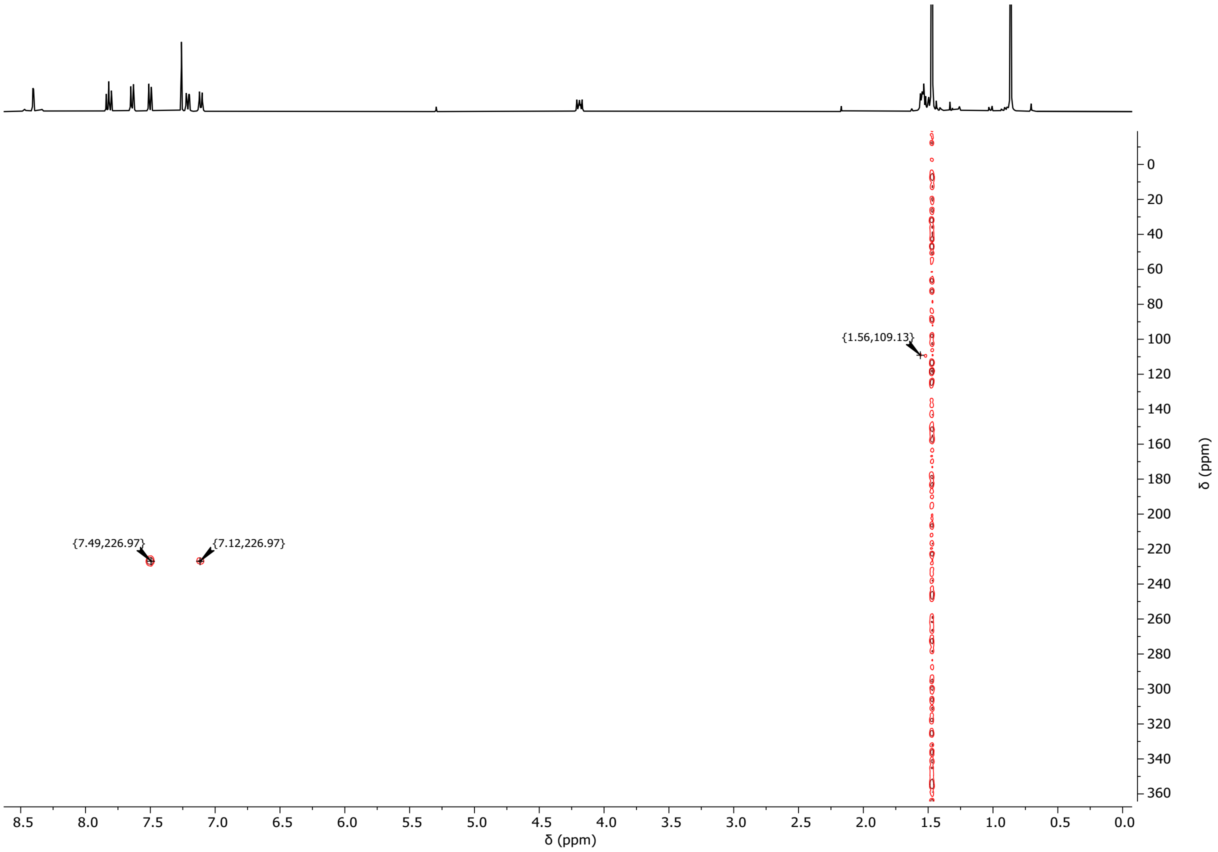

Figure S29: ^{1}H, ^{15}N-HMBC spectrum (400 MHz, 41 MHz, $CDCl_3$) of **Pt-*t*Bu**.

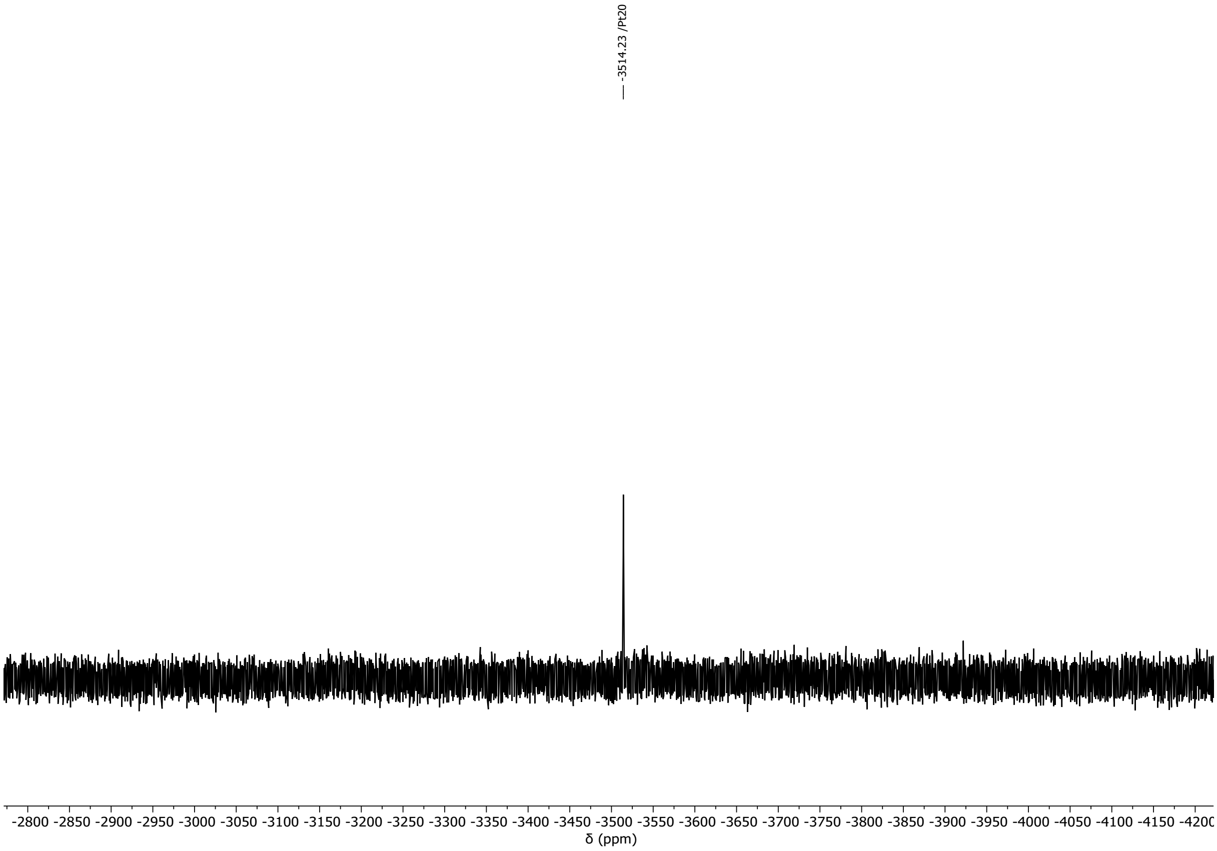

Figure S30: ^{195}Pt-NMR spectrum (86 MHz, $CDCl_3$) of **Pt-*t*Bu**.

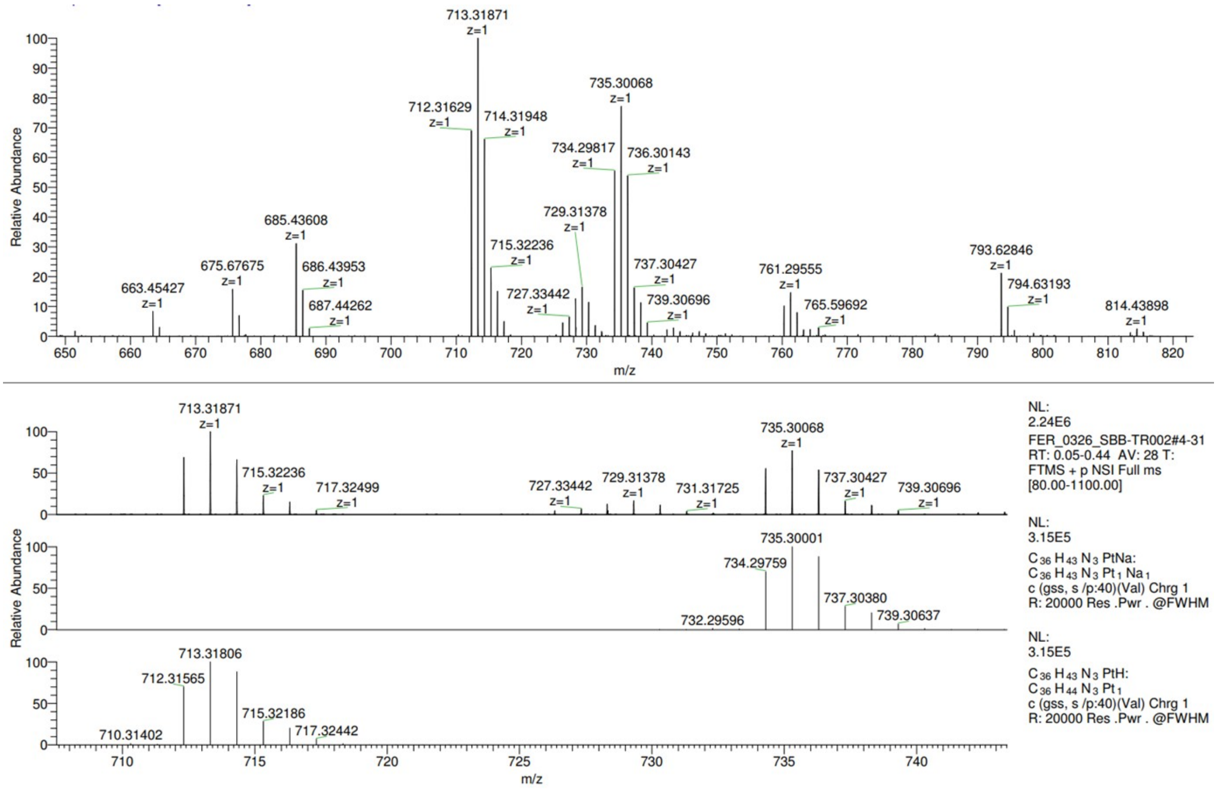

Figure S31: Mass spectrum of **Pt-*t*Bu** (top). Additional simulation of the [**Pt-*t*Bu**+H]$^+$ and [**Pt-*t*Bu**+Na]$^+$ adduct (bottom).

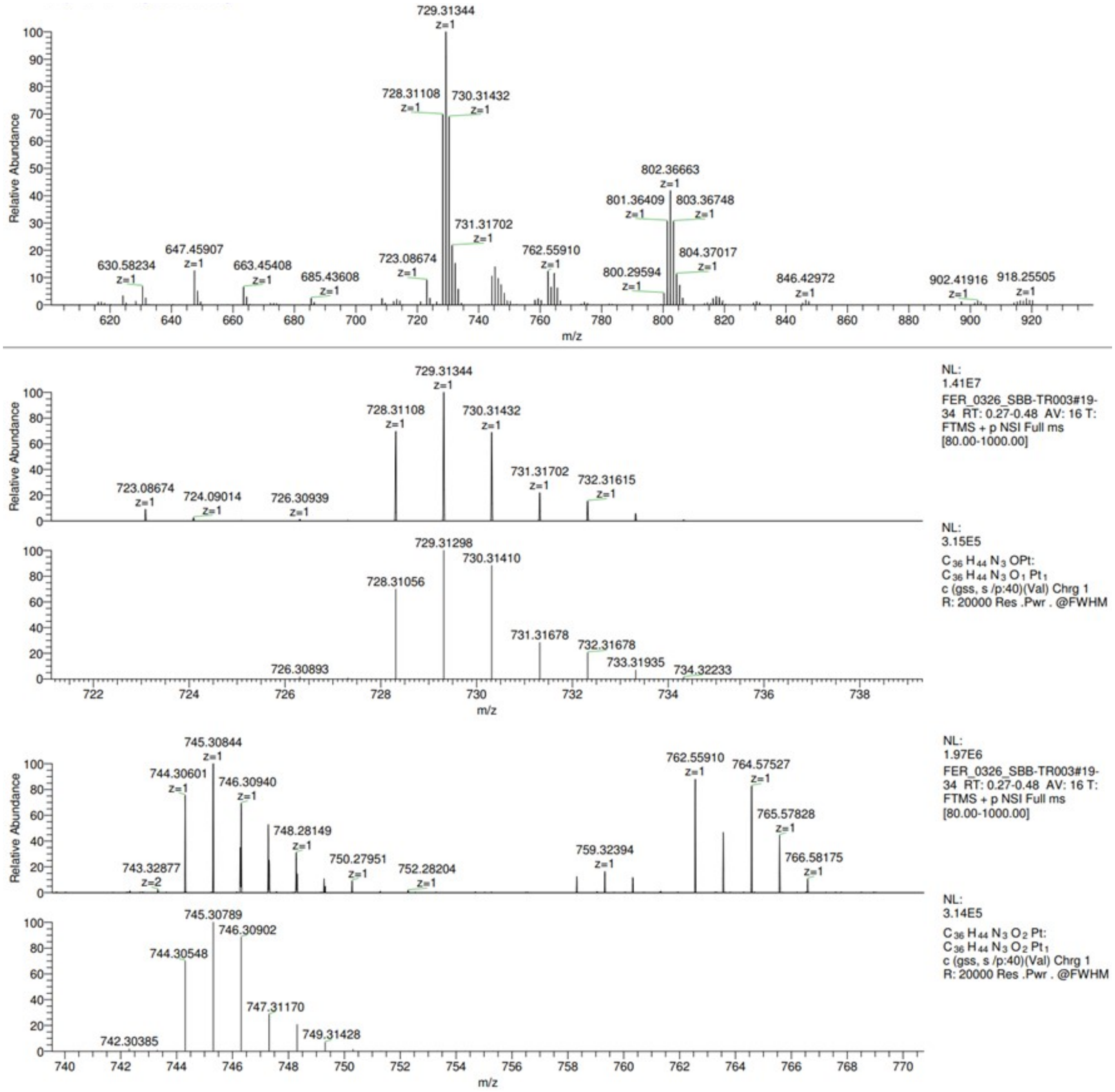

Figure S32: Mass spectrum of the DMF solution of **Pt-*t*Bu** and **Asc-Ac** after evaporation of the solvent under argon stream. Additional simulation of the [**Pt-*t*Bu**+OH]$^+$ and [**Pt-*t*Bu**+O_2H]$^+$ adduct.

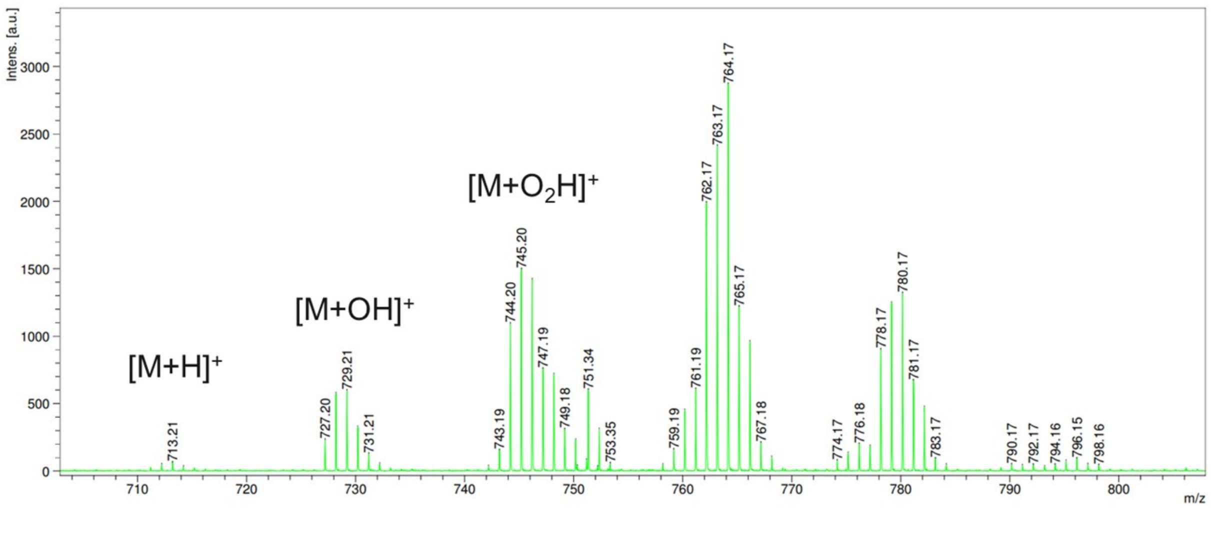

Figure S33: MALDI mass spectrum of the irradiated DMF solution (λ = 365 nm, 120 s) of **Pt-*t*Bu** and **Asc-Ac** after evaporation of the solvent under argon stream.

4. Photophysical characterization

Table S1: Complete photophysical data.

		λ_{ex} (nm)	λ_{em} (nm)	$\Phi_{L\ (air)}$ (± 2) (%)	$\Phi_{L\ (air)}$ (± 3) (%)	$\tau_{(air)}$ (ns)	$\tau_{(Ar)}$ (ns)
Fluid solution (298 K, DMF, 10^{-5} M)	**Pt-*t*Bu**	345, 370, 405	514, 549	< 2	59	124.6 ± 0.3	6895 ± 9

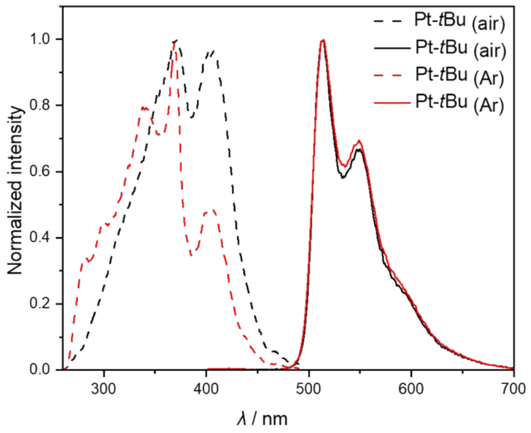

Figure S34: Excitation (dotted lines, λ_{em} = 514 nm) and emission spectra (solid lines, λ_{ex} = 370 nm) of **Pt-*t*Bu** in air-equilibrated (black) and Ar-purged (red) liquid DCM at room temperature. Normalized to highest intensity.

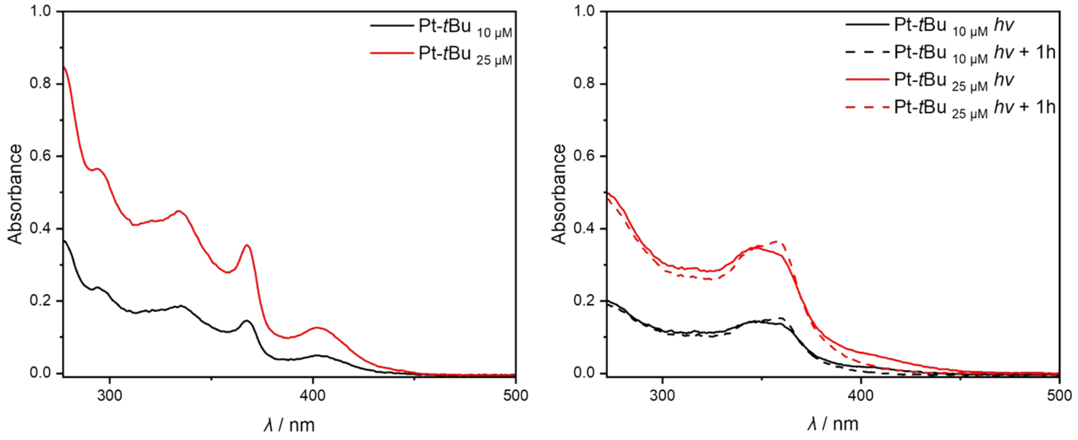

Figure S35: UV-vis absorption spectra of **Pt-*t*Bu** at two different concentrations (black lines: 10 µM; red lines: 25 µM) untreated (left), directly after irradiation (120 s) with λ = 365 nm (right solid) and 1 hour after irradiation (right dashed lines) in liquid DMF at room temperature.

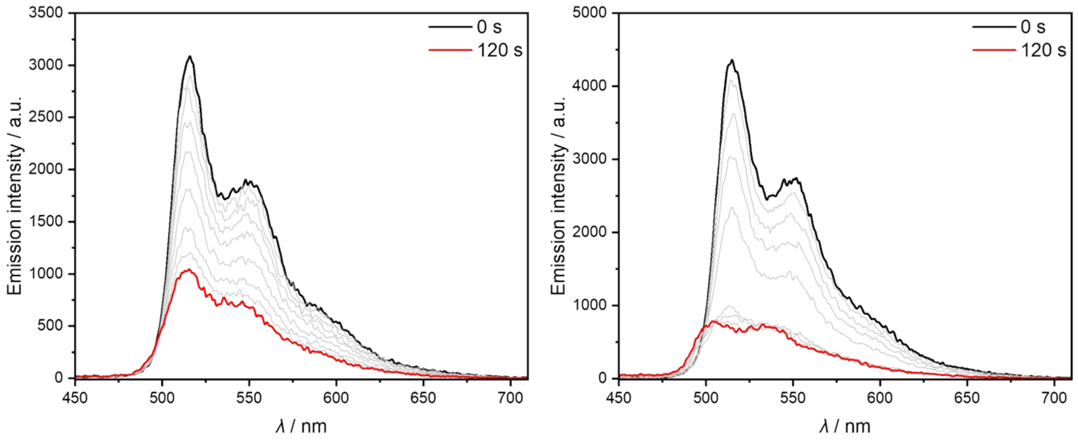

Figure S36: Emission spectra for two distinct **Pt-*t*Bu** concentrations (left: 10 µM; right: 25 µM) before and after different irradiation times with λ = 365 nm in air-equilibrated liquid DMF at room temperature.

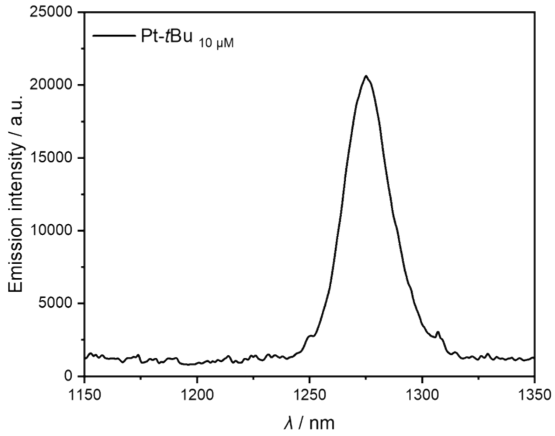

Figure S37: $^1O_2$ phosphorescence spectrum generated by **Pt-*t*Bu** (10 µM) in air-equilibrated liquid CD_2Cl_2 at room temperature.

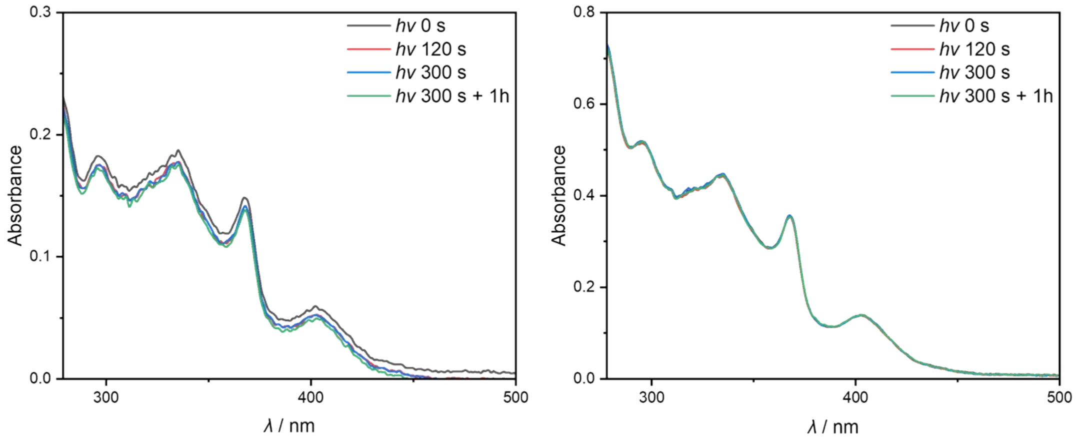

Figure S38: UV-vis absorption spectra for two distinct **Pt-*t*Bu** concentrations (left: 10 µM; right: 25 µM) before and after different irradiation times with λ = 365 nm in Ar-purged liquid DMF at room temperature.

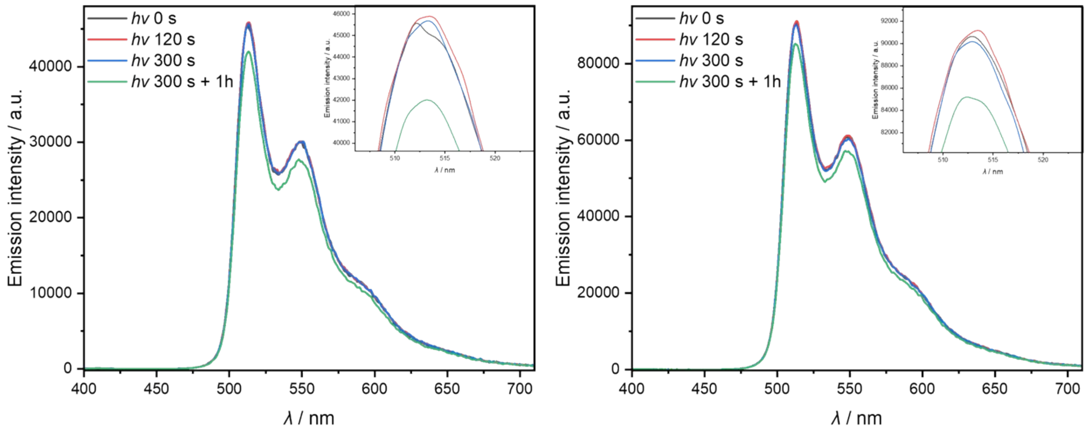

Figure S39: Emission spectra for two distinct **Pt-*t*Bu** concentrations (left: 10 µM; right: 25 µM) before and after different irradiation times with λ = 365 nm in Ar-purged liquid DMF at room temperature. Insets: Zoom of the spectra at the maximum.

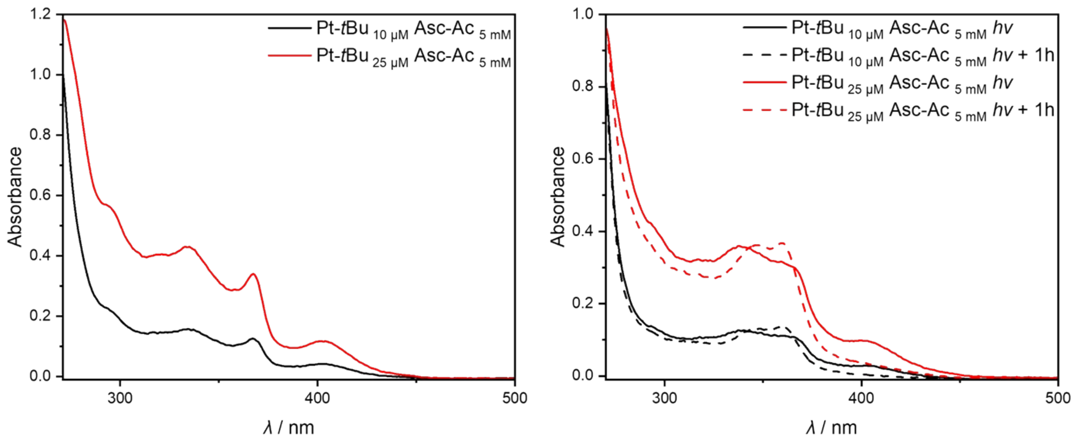

Figure S40: UV-vis absorption spectra of **Pt-*t*Bu** at two different concentrations (black lines: 10 µM; red lines: 25 µM) with **Asc-Ac** (5 mM) untreated (left), directly after irradiation (120 s) with λ = 365 nm (right solid) and 1 hour after irradiation (right dashed lines) in liquid DMF at room temperature.

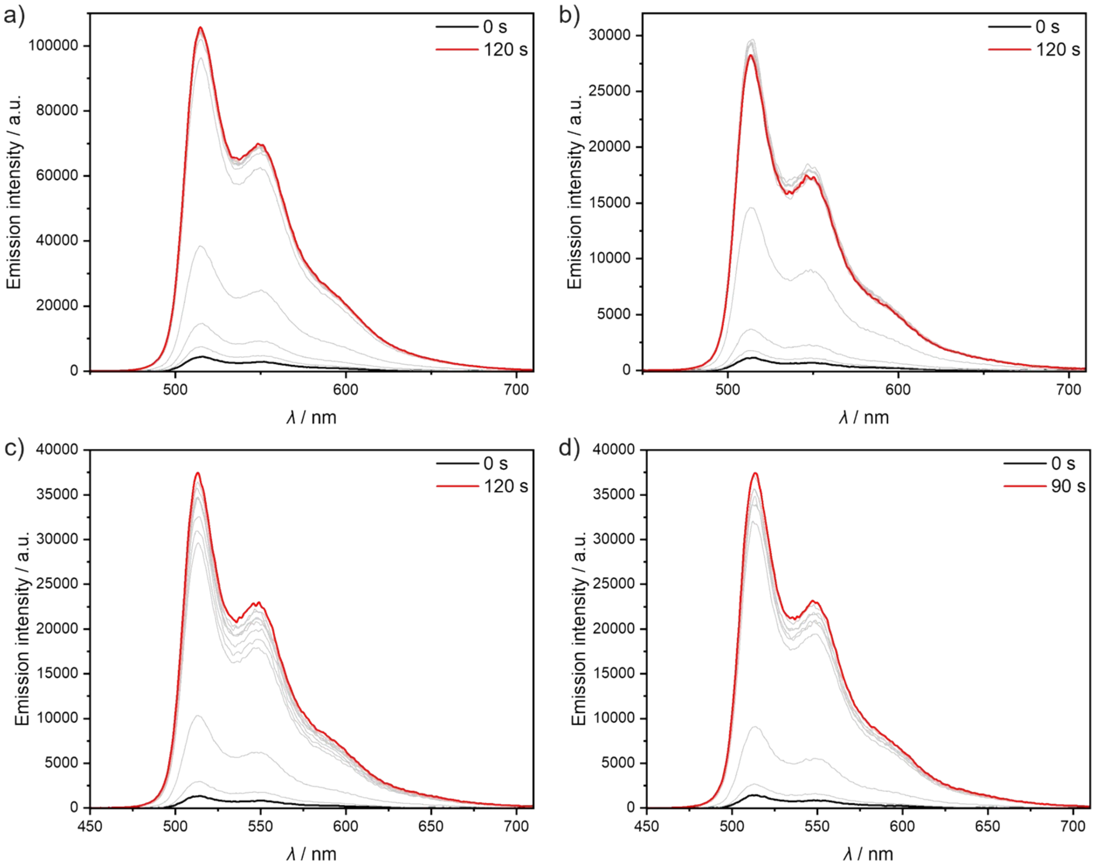

Figure S41: Emission spectra of **Pt-*t*Bu** with excess **Asc-Ac** at concentrations of: a) 10 µM/5 mM; b) 10 µM/10 mM; c) 25 µM/5 mM; d) 25 µM/10 mM. Spectra measured before and after consecutive irradiation intervals (a/b: 15 s; c/d: 10 s) with λ = 365 nm in liquid DMF at room temperature.

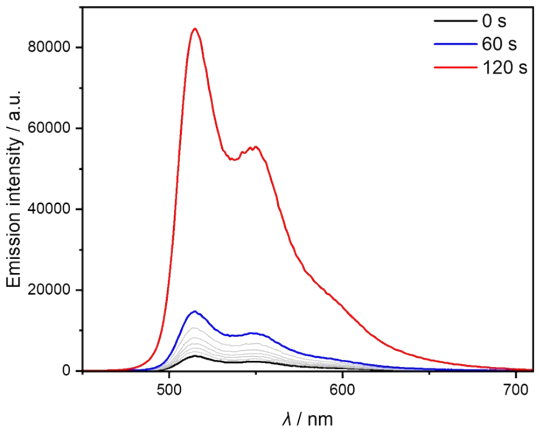

Figure S42: Emission spectrum of **Pt-*t*Bu** (10 µM) with **Asc-Ac** (5 mM). Spectra measured before and after consecutive irradiation intervals (6 x 10 s followed by 1 x 60 s) with λ = 365 nm in liquid DMF at room temperature.

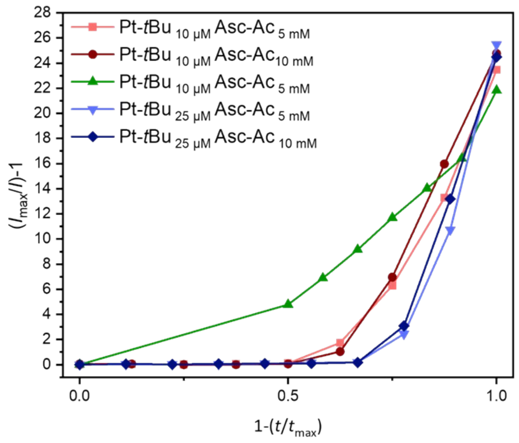

Figure S43: Plot of (I/I_{max})-1 vs. 1-(t/t_{max}) for different concentrations of **Pt-*t*Bu** and **Asc-Ac** at room temperature in liquid DMF.

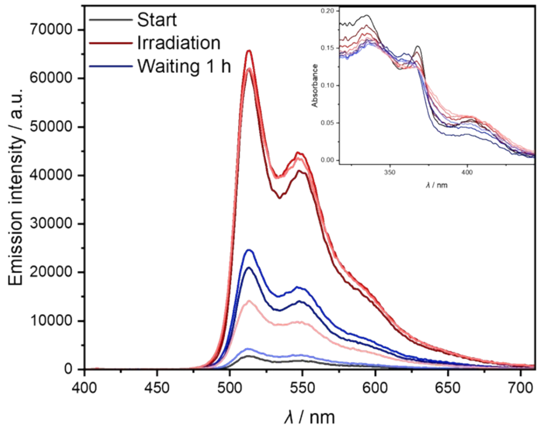

Figure S44: Emission spectra of **Pt-*t*Bu** (10 µM) with **Asc-Ac** (5 mM) after intervals of irradiation (red to lighter red) with λ = 365 nm (90 s) and waiting for 1 hour (blue to lighter blue) in liquid DMF at room temperature. Insets: Zoom of the corresponding UV-vis spectra in the region of 320 nm to 430 nm.

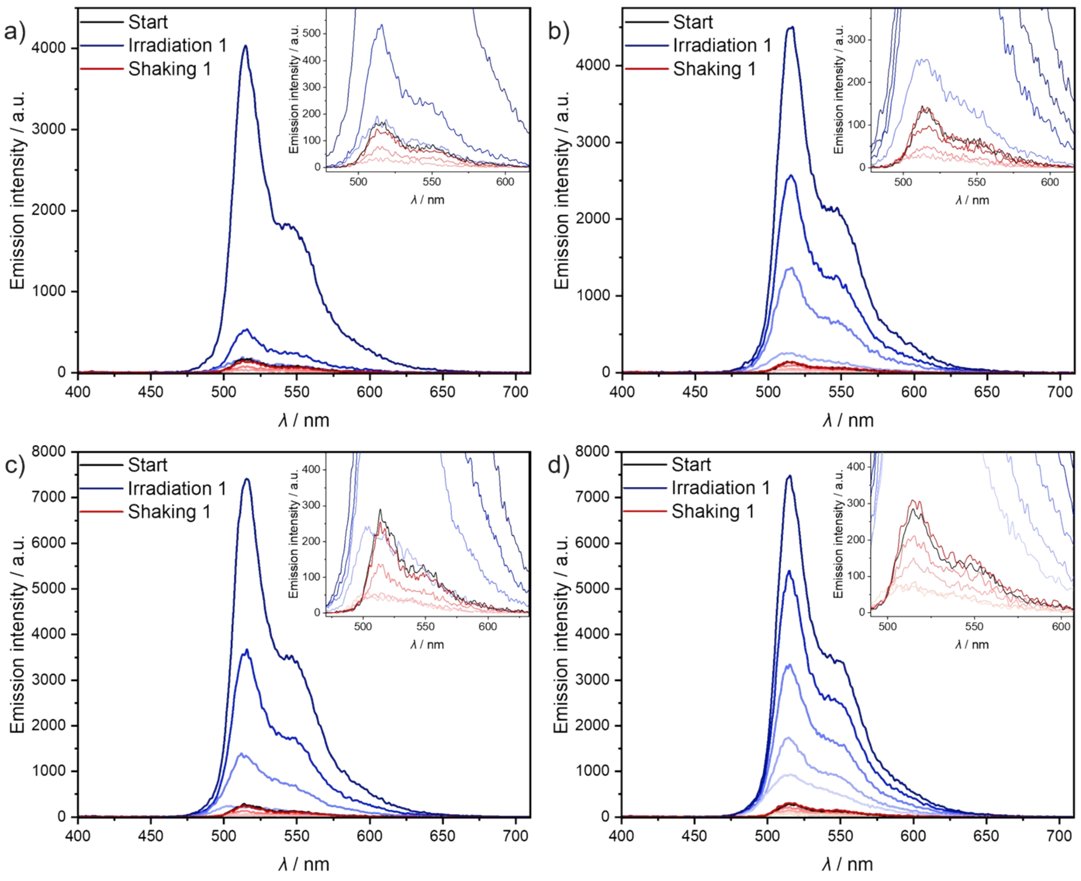

Figure S45: Emission spectra of **Pt-*t*Bu** with excess **Asc-Ac** at concentrations of: a) 10 µM/5 mM; b) 10 µM/10 mM; c) 25 µM/ 5 mM; d) 25 µM/10 mM. Spectra measured after various irradiation intervals (blue to lighter blue) with λ = 365 nm (90 s) and re-equilibration with air by shaking (red to lighter red) the sample in liquid DMF at room temperature. Insets: Zoom of the spectra at lower intensity.

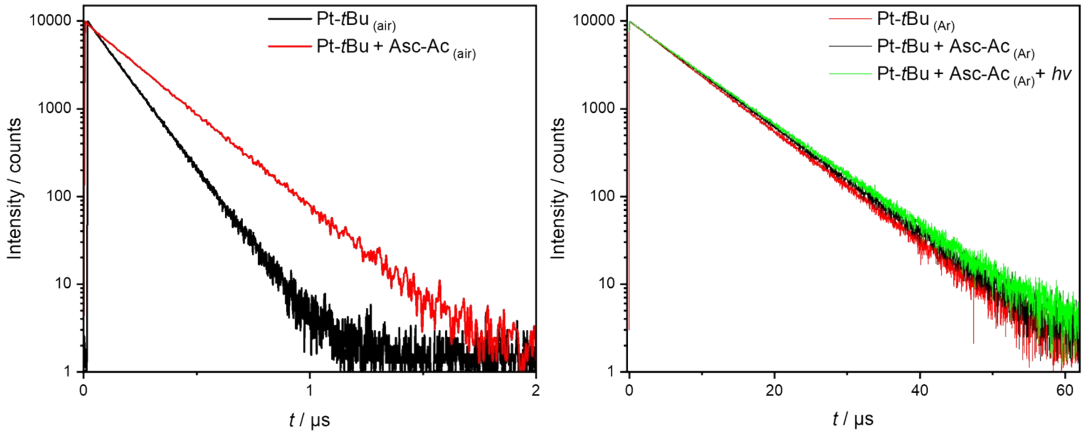

Figure S46: Comparison of luminescence lifetimes (monitored at 514 nm). Left: **Pt-*t*Bu** (25 µM) with (red) and without (black) **Asc-Ac** (10 mM). Right: **Pt-*t*Bu** (25 µM) with (black) and without (red) **Asc-Ac** (10 mM,) as well as after the irradiation process (green) in the photoreactor (λ = 365 nm) in Ar-purged liquid DMF at room temperature.

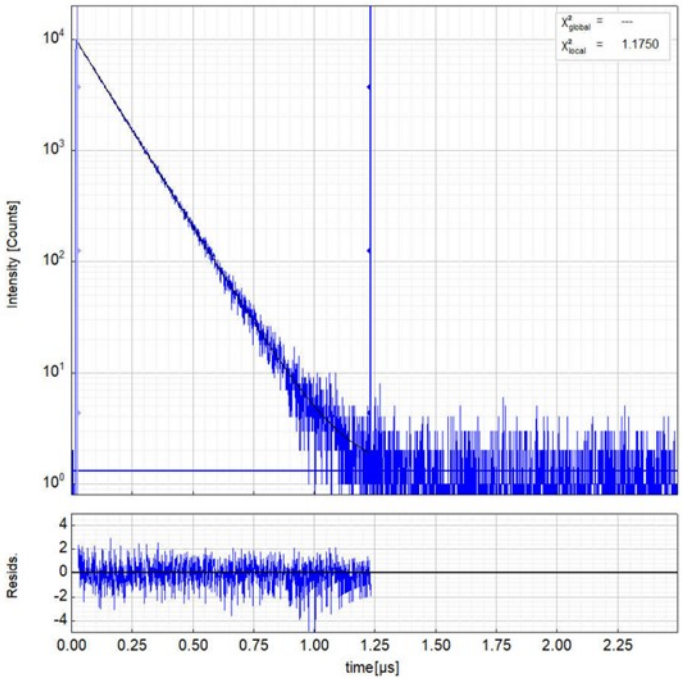

Parameter	Value	Δ	δ
A_1[kCnts/Chnl]	9.147	±0.031	0.3%
τ_1[ns]	124.58	±0.28	0.2%
I_1[kCnts]	1 139.5	±1.7	0.1%
A_{Rel1}[%]	100.0	---	---
I_{Rel1}[%]	100.0	---	---
$Bkgr_{Dec}$[kCnts]	0.0014	±0.0002	11%
τ_{AvInt}[ns]	124.58	±0.28	0.2%
τ_{AvAmp}[ns]	124.58	±0.28	0.2%

Figure S47: Left: Raw (experimental) time-resolved photoluminescence decay of **Pt-*t*Bu** in air-equilibrated liquid DCM at room temperature, including the residuals (λ_{ex} = 376.7 nm, λ_{em} = 514 nm). Right: Fitting parameters including pre-exponential factors and confidence limits.

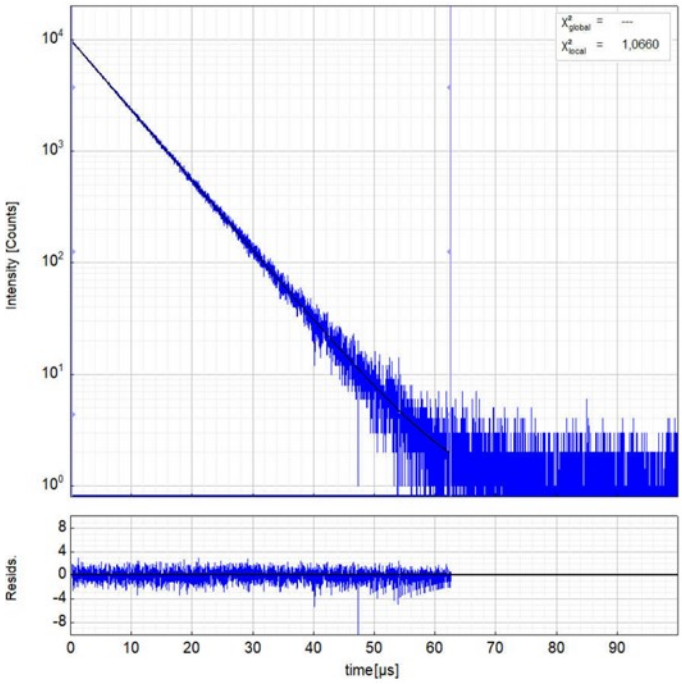

Parameter	Value	Δ	δ
A_1[kCnts/Chnl]	9,553	±0,022	0,2%
τ_1[ns]	6 895,1	±9,3	0,1%
I_1[kCnts]	4 116,7	±3,8	0,1%
A_{Rel1}[%]	100,0	---	---
I_{Rel1}[%]	100,0	---	---
$Bkgr_{Dec}$[kCnts]	0,0009	±0,0002	17%
τ_{AvInt}[ns]	6 895,1	±9,3	0,1%
τ_{AvAmp}[ns]	6 895,1	±9,3	0,1%

Figure S48: Left: Raw (experimental) time-resolved photoluminescence decay of **Pt-*t*Bu** in Ar-purged liquid DCM at room temperature, including the residuals (λ_{ex} = 376.7 nm, λ_{em} = 514 nm). Right: Fitting parameters including pre-exponential factors and confidence limits.

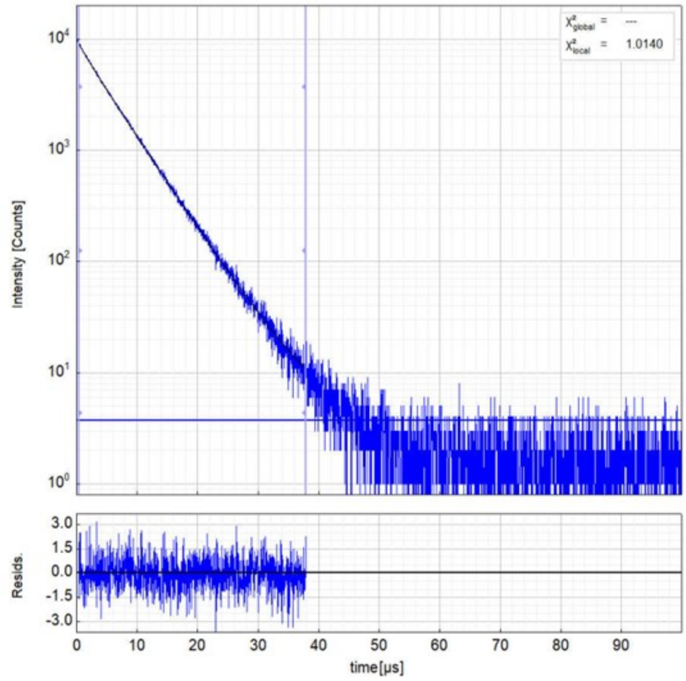

Parameter	Value	Δ	δ
A_1[kCnts/Chnl]	8.07	±0.36	4.4%
τ_1[ns]	5 332	±64	1.2%
I_1[kCnts]	1 344	±44	3.2%
A_{Rel1}[%]	88.9	±3.5	3.9%
I_{Rel1}[%]	95.4	±3.1	3.2%
A_2[kCnts/Chnl]	1.02	±0.31	31%
τ_2[ns]	2 100	±570	27%
I_2[kCnts]	67	±44	66%
A_{Rel2}[%]	11.2	±3.5	31%
I_{Rel2}[%]	4.7	±3.1	66%
$Bkgr_{Dec}$[kCnts]	0.0037	±0.0007	18%
τ_{AvInt}[ns]	5 179.3	±8.0	0.2%
τ_{AvAmp}[ns]	4 971	±26	0.5%

Figure S49: Left: Raw (experimental) time-resolved photoluminescence decay of **Pt-*t*Bu** (10 µM) with **Asc-Ac** (5 mM) after irradiation with 365 nm (120 s) in air-equilibrated liquid DMF at room temperature, including the residuals (λ_{ex} = 376.7 nm, λ_{em} = 514 nm). Right: Fitting parameters including pre-exponential factors and confidence limits.

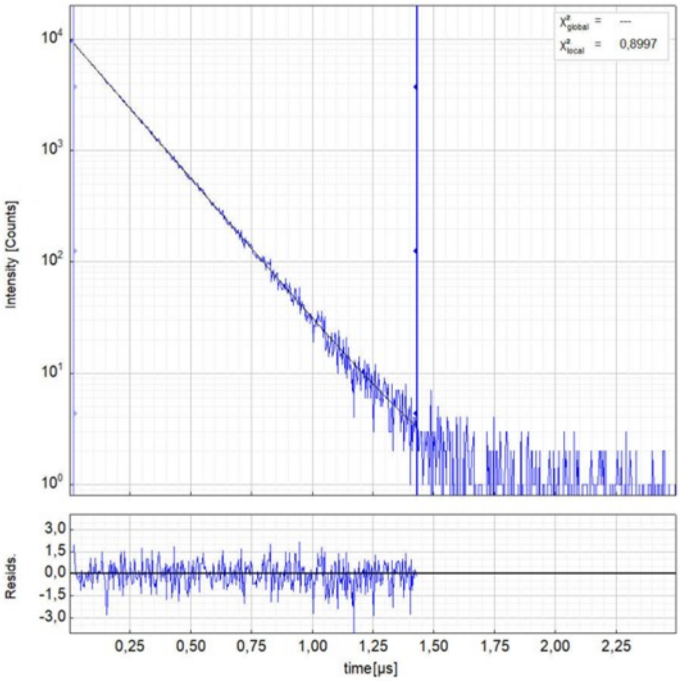

Parameter	Value	Δ	δ
A_1[kCnts/Chnl]	9,130	±0,031	0,3%
τ_1[ns]	172,18	±0,32	0,2%
I_1[kCnts]	392,97	±0,95	0,2%
A_{Rel1}[%]	100,0	---	---
I_{Rel1}[%]	100,0	---	---
$Bkgr_{Dec}$[kCnts]	0,0008	±0,0005	64%
τ_{AvInt}[ns]	172,18	±0,32	0,2%
τ_{AvAmp}[ns]	172,18	±0,32	0,2%

Figure S50: Left: Raw (experimental) time-resolved photoluminescence decay of **Pt-*t*Bu** (25 µM) with **Asc-Ac** (10 mM) in air-equilibrated liquid DMF at room temperature, including the residuals (λ_{ex} = 376.7 nm, λ_{em} = 514 nm). Right: Fitting parameters including pre-exponential factors and confidence limits.

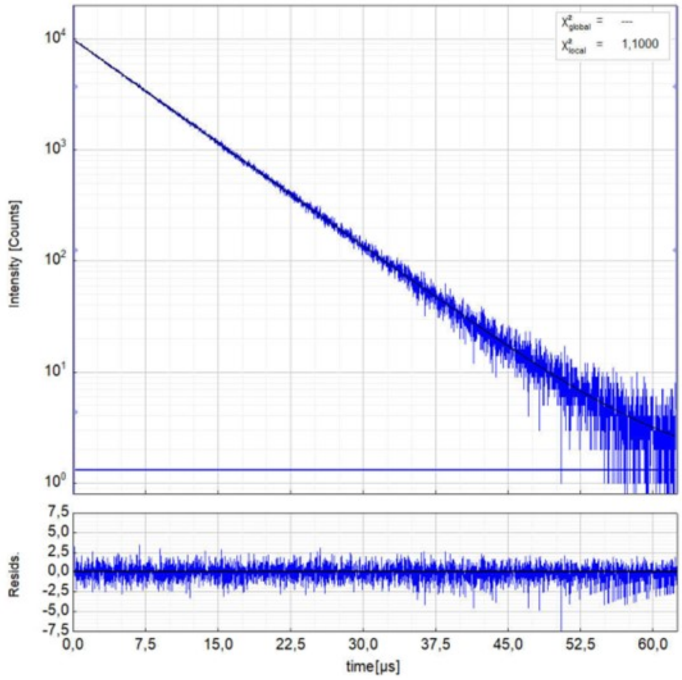

Parameter	Value	Δ	δ
A_1[kCnts/Chnl]	9,7223	±0,0041	0,0%
τ_1[ns]	6 989,6	±2,9	0,0%
I_1[kCnts]	4 247,2	±1,9	0,0%
A_{Rel1}[%]	100,0	---	---
I_{Rel1}[%]	100,0	---	---
$Bkgr_{Dec}$[kCnts]	0,0014	±0,0001	4,8%
τ_{AvInt}[ns]	6 989,6	±2,9	0,0%
τ_{AvAmp}[ns]	6 989,6	±2,9	0,0%

Figure S51: Left: Raw (experimental) time-resolved photoluminescence decay of **Pt-*t*Bu** (25 µM) with **Asc-Ac** (10 mM) in Ar-purged liquid DMF at room temperature, including the residuals (λ_{ex} = 376.7 nm, λ_{em} = 514 nm). Right: Fitting parameters including pre-exponential factors and confidence limits.

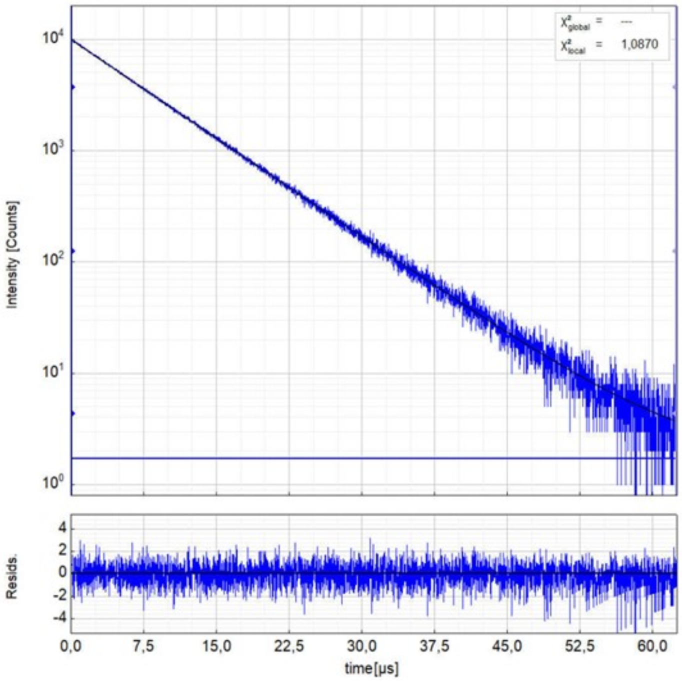

Parameter	Value	Δ	δ
A_1[kCnts/Chnl]	9,836	±0,015	0,1%
τ_1[ns]	7 330,8	±7,8	0,1%
I_1[kCnts]	4 506,2	±4,4	0,1%
A_{Rel1}[%]	100,0	---	---
I_{Rel1}[%]	100,0	---	---
$Bkgr_{Dec}$[kCnts]	0,0018	±0,0002	8,8%
τ_{AvInt}[ns]	7 330,8	±7,8	0,1%
τ_{AvAmp}[ns]	7 330,8	±7,8	0,1%

Figure S52: Left: Raw (experimental) time-resolved photoluminescence decay of **Pt-*t*Bu** (25 µM) with **Asc-Ac** (10 mM) after irradiation with 365 nm (90 s) in Ar-purged liquid DMF at room temperature, including the residuals (λ_{ex} = 376.7 nm, λ_{em} = 514 nm). Right: Fitting parameters including pre-exponential factors and confidence limits.

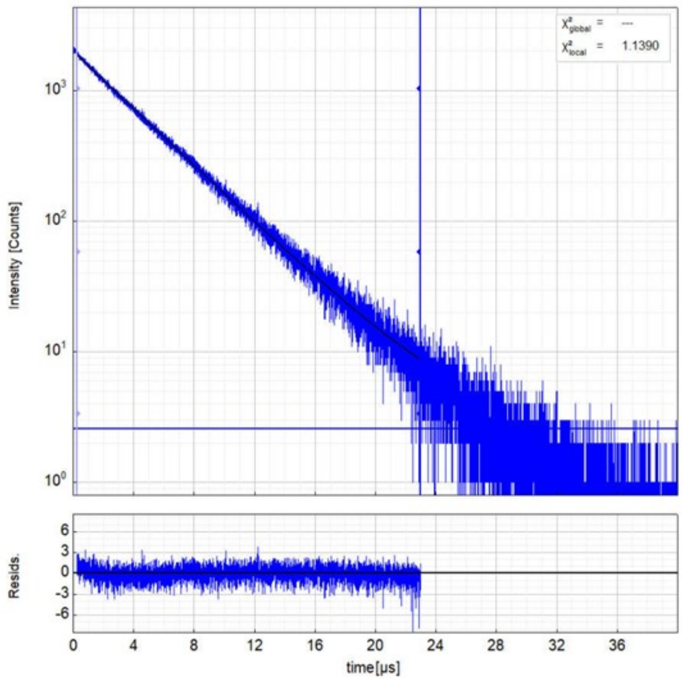

Parameter	Value	Δ	δ
A_1[kCnts/Chnl]	1.8694	±0.0045	0.2%
τ_1[ns]	3 980.2	±5.6	0.1%
I_1[kCnts]	1 860.2	±1.9	0.1%
A_{Rel1}[%]	100.0	---	---
I_{Rel1}[%]	100.0	---	---
$Bkgr_{Dec}$[kCnts]	0.0026	±0.0003	11%
τ_{AvInt}[ns]	3 980.2	±5.6	0.1%
τ_{AvAmp}[ns]	3 980.2	±5.6	0.1%

Figure S53: Left: Raw (experimental) time-resolved photoluminescence decay of **Pt-*t*Bu** (25 µM) with **Asc-Ac** (10 mM) after irradiation with 365 nm (90 s) in air-equilibrated liquid DMF at room temperature, including the residuals (λ_{ex} = 376.7 nm, λ_{em} = 514 nm). Right: Fitting parameters including pre-exponential factors and confidence limits.

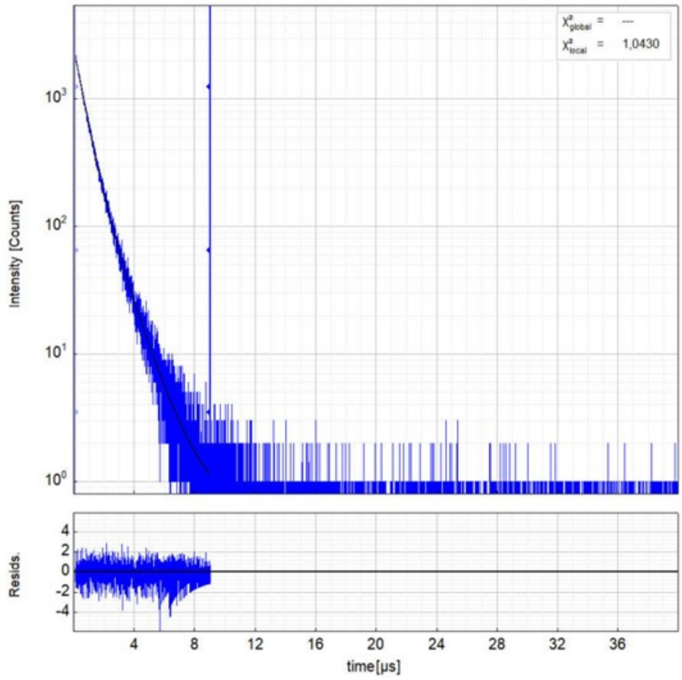

Parameter	Value	Δ	δ
A_1[kCnts/Chnl]	0,443	±0,074	17%
τ_1[ns]	1 309	±59	4,5%
I_1[kCnts]	145	±17	11%
A_{Rel1}[%]	20,4	±3,4	16%
I_{Rel1}[%]	35,4	±4,1	11%
A_2[kCnts/Chnl]	1,732	±0,072	4,1%
τ_2[ns]	611	±15	2,3%
I_2[kCnts]	265	±17	6,3%
A_{Rel2}[%]	79,7	±3,4	4,2%
I_{Rel2}[%]	64,7	±4,1	6,3%
$Bkgr_{Dec}$[kCnts]	0,0007	±0,0003	43%
τ_{AvInt}[ns]	857,4	±4,8	0,6%
τ_{AvAmp}[ns]	752,5	±5,2	0,7%

Figure S54: Left: Delayed (two min) raw (experimental) time-resolved photoluminescence decay of **Pt-*t*Bu** (25 µM) with **Asc-Ac** (10 mM) after irradiation with 365 nm (90 s) in air-equilibrated liquid DMF at room temperature, including the residuals (λ_{ex} = 376.7 nm, λ_{em} = 514 nm). Right: Fitting parameters including pre-exponential factors and confidence limits.

5. Broadening the scope to other metal complexes

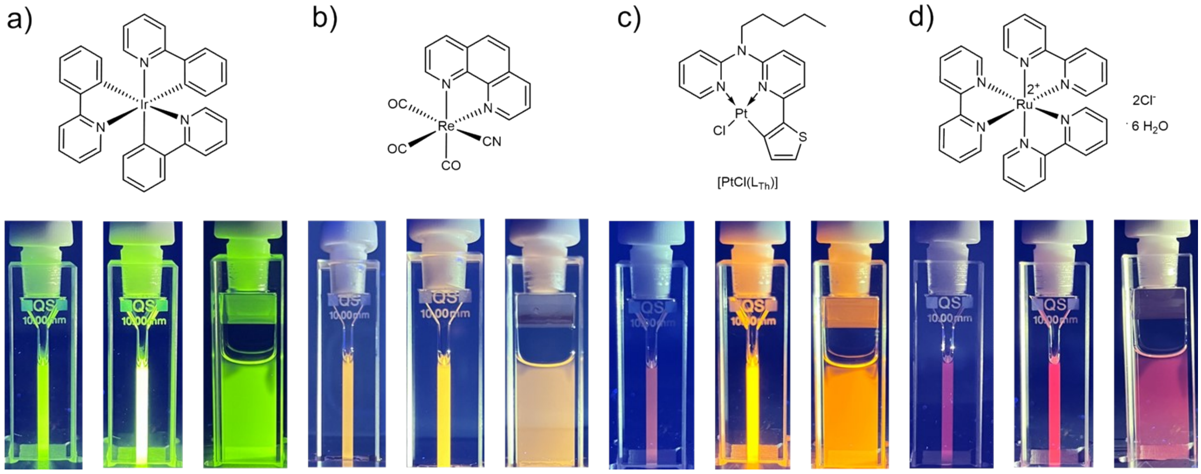

Supplementary Figure 55: Structural formulae of *fac*-tris-(2-phenylpyridin)-iridium(III)[6] (a), tricarbonyl(cyanido)(1,10-phenanthroline)rhenium(I) (b),[7] [**PtCl(l$_{Th}$)**][4] (c) and tris(2,2′-bipyridyl)ruthenium(II) dichloride hexahydrate[5] (d). Shown are pictures under UV-light of the cuvettes with solutions of each complex (25 µM) containing **Asc-Ac** (10 mM) in liquid DMF before and after the full irradiation process (λ = 365 nm). Pictures on the right demonstrate the diffusion of fresh oxygen after irradiation.

6. Supplementary References

1. G. R. Fulmer, A. J. M. Miller, N. H. Sherden, H. E. Gottlieb, A. Nudelman, B. M. Stoltz, J. E. Bercaw, K. I. Goldberg, *Organometallics*, 2010, **29**, 2176-2179.
2. E.-X. Zhang, D.-X. Wang, Z.-T. Huang, M.-X. Wang, *The Journal of Organic Chemistry*, 2009, **74**, 8595-8603.
3. S. Buss, M. V. Cappellari, A. Hepp, J. Kösters, C. A. Strassert, *Chemistry*, 2023, **5**, 1243-1255.
4. S. Buss, L. Geerkens, I. Maisuls, J. Kösters, N. Bäumer, G. Fernández, C. A. Strassert, *Organometallics*, 2024, doi.org/10.1021/acs.organomet.3c00540.
5. J. A. Broomhead, C. G. Young, P. Hood in *Inorg. Synth*., 1990, **28**, 338–340.
6. S. Lamansky, P. Djurovich, D. Murphy, F. Abdel-Razzaq, R. Kwong, I. Tsyba, M. Bortz, B. Mui, R. Bau, M. E. Thompson, *Inorg. Chem.*, 2001, **40**, 1704-1711.
